# Trends in incidence and mortality of early-onset cancer in Germany between 1999 and 2019

**DOI:** 10.1007/s10654-024-01134-4

**Published:** 2024-05-31

**Authors:** Dina Voeltz, Kira Baginski, Claudia Hornberg, Annika Hoyer

**Affiliations:** 1 Biostatistics and Medical Biometry, Medical School OWL, Bielefeld University, Bielefeld, Germany; 2 Department of Statistics, Ludwig-Maximilians-University Munich, Munich, Germany; 3 Department of Environmental Health Sciences, Medical School OWL, Bielefeld University, Bielefeld, Germany

**Keywords:** Cancer, Germany, Incidence, Mortality

## Abstract

Evidence on the recent temporal trend in the incidence and mortality of early-onset cancer, i.e., cancer diagnosed at ages of < 50 years, in Germany is scarce. To estimate the temporal trend in the incidence and mortality of early-onset cancer in Germany between 1999 and 2019. Input data were obtained from the Centre for Cancer Registry Data (Zentrum für Krebsregisterdaten, ZfKD). The analysis comprised all ages until 50 years and all types of cancer classified by the International Classification of Diseases (ICD-10)-codes C00-C97 (excl. C44). Temporal trends were estimated using negative binomial regression, differentiated by sex and cancer type. Between 1999 and 2019 in Germany, we observed stable or slightly increasing trends (0% and 1%) in the incidence of all early-onset cancers combined (C00-C97) for men and women, respectively, and strict declines in the mortality for both, men and women (-2% and − 3%). However, the trends differ largely with respect to sex and the individual cancer types. Early-onset cancer should be closely monitored to see whether stable and decreasing trends in the incidence and mortality continue. Knowing that despite decreasing incidence, the prevalence of a disease can rise due to their interplay with mortality, we recommend to maintain precise surveillance, efforts in prevention and early detection, as well as appropriate investments into healthcare resources, research and development.

**Supplementary Information:**

The online version contains supplementary material available at 10.1007/s10654-024-01134-4.

## Introduction

Worldwide, the global cancer burden continues to increase [1–3]. Although many cancers can be effectively treated if detected early, cancer is a leading cause of death globally and continues to be the main disease hindering life expectancy [3, 4]. The multifactorial disease is generally more prevalent in people older than 50 years of age, but a variety of studies indicate an increase in the incidence of early-onset cancer (i.e., people younger than 50 years) [1, 2]. Compared to later-onset cancer, early-onset cancer is associated with a higher burden of personal, societal, clinical and economic ramifications [1, 4]. As such, early-onset cancer is typically more aggressive and associated with poorer survival outcomes compared to later-onset cancer [5], can lead to a higher number of depressions at younger ages [5], and is related to a higher risk of additional and/or long-term health problems such as infertility, cardiovascular disease and secondary cancers [5]. However, little is known about the epidemiology and disease burden of early-onset cancer. Only recently, Lin et al. [4] and Zhao et al. [1] systematically evaluated the global burden of 29 early-onset cancers (i.e., 14 to 49 years of age at the time of diagnosis) between 1990 and 2019. In 2019, the incidence of early-onset cancer was estimated at 3.26 mio cases globally, indicating a 79.1% increase compared to 1990 (1.82 mio) [1]. By 2030, the number of new cancer cases and associated deaths are predicted to increase by 31% and 20%, respectively, with those over 40 years of age at highest risk [1]. Further, the analyses of Lin et al. [4] and Zhao et al. [1] revealed significant differences in the incidence and mortality related to early-onset cancer across regions, countries and cancer types. Overall, countries with higher sociodemographic index were particularly affected and exhibited the highest incidence of early-onset cancer compared to those with a lower sociodemographic index [1, 2, 4]. The main causes of this increasing trend remain unclear. Attempts to explain the growing burden are related to increasing uptake of early screening interventions and exposure to risk factors in early life or young adulthood, as well as to (western) lifestyle, dietary risk factors, alcohol consumption and tobacco use [1, 2, 5].

In Germany, evidence on the recent temporal trend in the incidence and mortality of early-onset cancer in Germany is scarce. Although Lin et al. [4] and Zhao et al. [1] include Germany in their studies, their focus was on examining global trends and disease burden of early-onset cancer rather than making more fine-grained statements on regional and national levels. Further, both studies rely on input data from the Global Burden of Disease (GBD) study which synthesizes a large number of input sources to estimate mortality, causes of death and illness, and risk factors. More precisely, with regard to German cancer data, their input data includes information from six (instead of all 16) federal states in Germany. Consequently, the accuracy of their estimation is compromised and largely depends on the quality and quantity of cancer registry data in different countries that is combined in the GBD database. In the light of (i) recent criticism of some of the methodological aspects of the GBD database [6, 7], (ii) evidence that the GBD methodology might have led to inaccurate estimates in some analyses [6], (iii) the limited availability of nationwide cancer registry data in Germany only since 1999 [8, 9], and (iv) according to the German Cancer Research Centre (Deutsches Krebsforschungszentrum, DKFZ) [8], the findings of Zhao et al. [1] cannot be directly transferred to Germany. However, the discussed global findings related to early-onset cancer are particularly perturbing, knowing that Germany has a high socioeconomic index with more than half of the population being younger than 50 years [10]. Further, the country meets the main risk factors, for instance in terms of its Western lifestyle and its high prevalence of overweight and obesity [11]. Therefore, the main purpose of this study is to analyse the sex-, age- and cancer-specific temporal trend in the incidence and mortality for people aged younger than 50 years between 1999 and 2019 in Germany.

## Methods

### Input data

Each federal state in Germany collects epidemiological cancer data in a registry. With the passing of the Federal Cancer Registry Data Act (Bundeskrebsregisterdatengesetz, BKRG) in Germany in 2009, these registries are obliged to transmit their data to a nationwide database maintained by the Centre for Cancer Registry Data (Zentrum für Krebsregisterdaten, ZfKD) of the Robert Koch Institute (RKI) [9, 12]. By now, this nationwide registry has reached almost full completeness, covering over 95% of all cancer cases in Germany [9, 12–14]. From this database, we obtained input data for the disease-specific mortality, i.e., the mortality of individuals with cancer, and the incidence of cancer for each year between 1999 and 2019. The data are reported as crude rate per 100,000 by year, sex and 5-year age-groups (from 0 until 85 years) and is differentiated by cause of death according to the International Classification of Diseases (ICD). The German cancer registry provides all incidence and mortality data for the different cancer types with consecutive ICD-10 codes on aggregated level for 18 different subtypes (see supplementary information, Table S1). The analysis was conducted for each subgroup of cancer separately. As implied by definition of early-onset cancer, we included ages between 0 and 50 years only. For simplicity, we will use the terms “women” and “men” although the considered age range includes boys and girls as well as women and men.

The analysis only required published aggregated data on population level. All data used were well prepared and pre-processed, i.e. no missing or incomplete data had to be taken care of. Patient and public engagement/ involvement was not applicable or relevant to our proposed project.

### Statistical methods

As there remains debate about the optimal statistical method for analysing and predicting potential temporal trends in cancer-registry data [15], we applied three different statistical methods. In our main analysis, we quantified trends in the incidence and mortality using negative binomial regression as for the reasons given below. As sensitivity analyses, temporal trends were computed via Poisson regression and Joinpoint regression. Negative binomial and Poisson regression were conducted using the statistical software R, version 4.3.1 [16]. The source code for R is publicly available on Zenodo [17]. Joinpoint regression models were fitted using the National Cancer Institute’s (NCI) Joinpoint software [18–20]. We used a log-linear model as the resulting rates provide the change at a constant percent per year, while the linear model returns constant fixed amounts. Hence, results are more easily comparable across different cohorts (e.g., sex, cancer type). To align with the methodology across published literature [21], we adopted the usage of a maximum number of three joinpoints. For each model, the optimal number of joinpoints was chosen as recommended by the software which is based upon the Weighted Bayesian Information Criterion. We report the Average Annual Percent Change (AAPC).

For all methods, the trend estimation was conducted separately for each sex and for the 18 early-onset cancer groups as well as for all types of cancer combined (i.e., ICD-10 C00-C97 excl. C44).

## Results

### Incidence

**Fig. 1:**
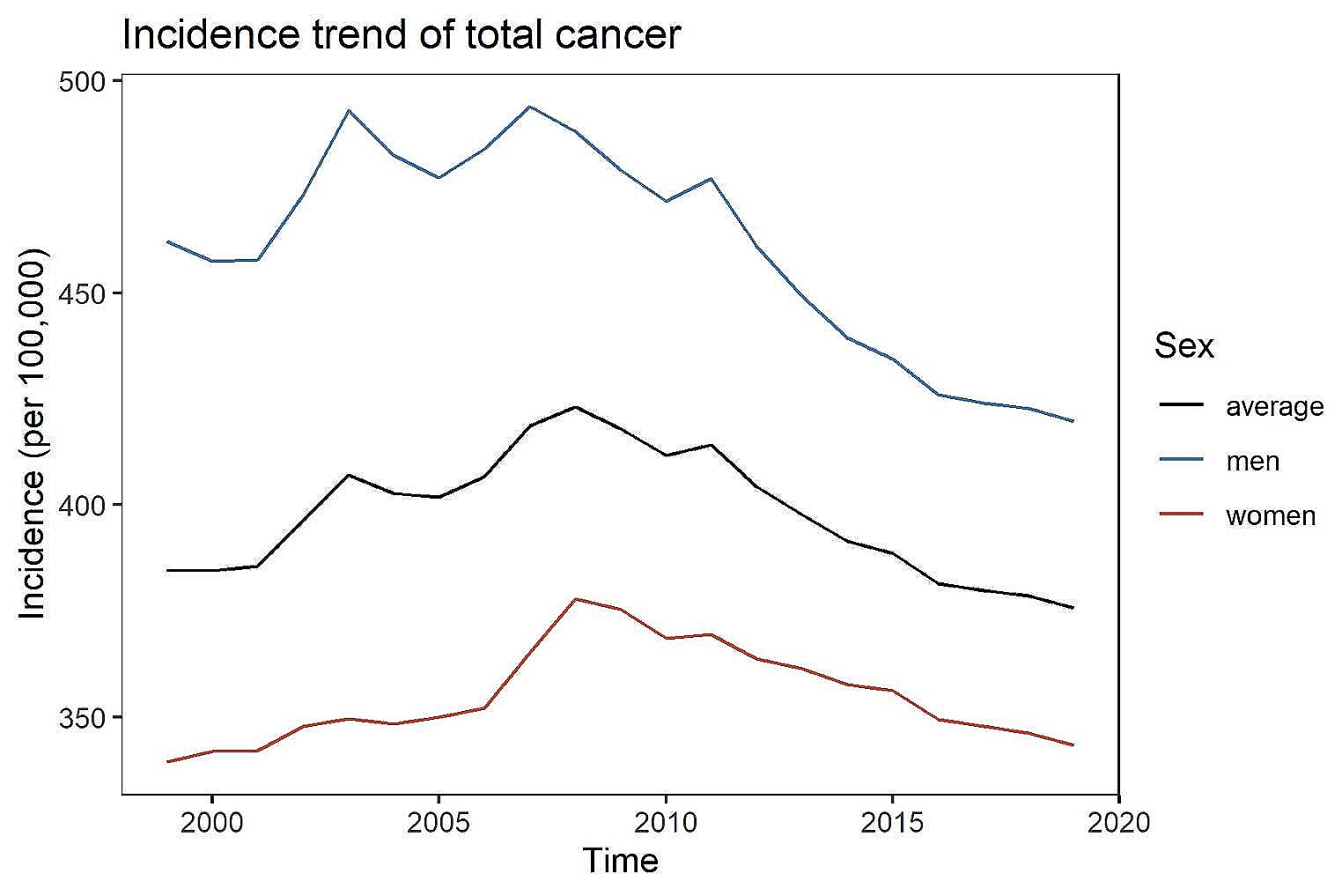
Age-standardized incidence rate (per 100,000) of all early-onset cancers (C00-C97) combined between 1999 and 2019

In Germany in 2019, the age-standardised incidence of all early-onset cancer types combined was 375.8 per 100,000, a 2% decrease compared to 1999 (384.6 per 100,000) (see Fig. 1). It is noticeable that, until about 2010, there is a strict upward trend in the incidence. Nonetheless, thereafter until 2019, the incidence showed a downward trend for both men and women. Overall between 1999 and 2019, the incidence of all early-onset cancers together in Germany is estimated to increase by 1% (95% confidence interval [0%; 1%]) per year for women (see Table S2, Figure S7). For men, the analysis indicates a stable incidence (0% change [95% CI: 0%; 1%]) over time (see Table S2, Figure S6). Generally, the incidence of all early-onset cancer (C00-C97 combined) is considerably lower than the incidence of late-onset cancer (i.e., cancer diagnosed in people > 50 years) (see Figure S13). However, despite the substantial difference in the incidence in absolute terms, the incidence trend developed relatively similar comparing early-onset vs. late-onset cancer.

The incidence of early-onset cancer (C00-C97 combined) is higher in men than in women (Fig. 1). This difference becomes more pronounced with increasing age. It is estimated that for an additional year of age, the incidence of all cancer types combined increases on average by 7% [95% CI: 7%; 7%] for men and by 9% [95% CI: 9%; 10%] for women (see Table S2, Figure S9 and S10). This holds true when considering the cancer types in a more differentiated manner. With only a few exceptions where the incidence remains stable with changing age, the incidence substantially rises with higher ages (see Table S2, Fig. 1, Figures S1 and S2). The sharpest increase in the incidence of early-onset cancer among men is estimated for C90 (Multiple myeloma and malignant plasma cell neoplasms) with 19% [95% CI: 15%; 23%] increase in the incidence for every additional year of age. For women, the strongest average increase in the incidence in relation to higher age (+ 19%) is seen for C50 (Malignant neoplasm of breast, [18%; 19%]) and C90 (Multiple myeloma and malignant plasma cell neoplasms, [95% CI: 15%; 23%]) (see Table S2).

**Fig. 2:**
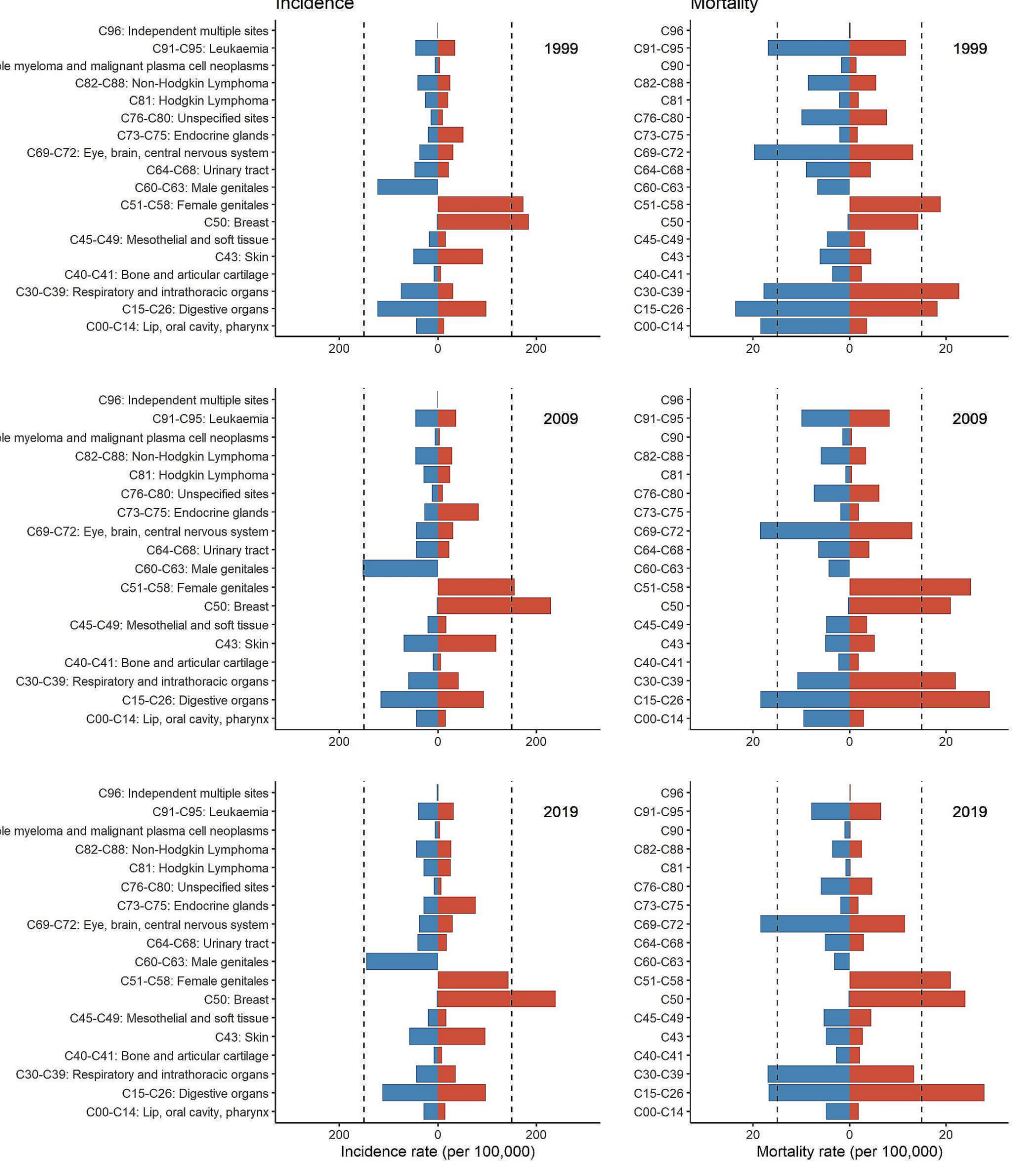
Incidence and mortality rate in 1999, 2009 and 2019 by sex and cancer type

Among all cancer types, early-onset cancer of digestive organs (C15-C26), breast cancer (C50) and cancer of female genital organs (C51-C58) had the highest incidence among women for all years between 1999 and 2019 (see Fig. 2). For men, the early-onset cancers with the greatest disease burden were cancers of digestive organs (C15-C26), respiratory and intrathoracic organs (C30-C39) and male genital organs (C60-C63). Among women, the incidence of early-onset cancers of female genital organs (C51-C58) and ill-defined, secondary and unspecified sites (C76-C89) showed decreases of 1% ([95% CI: -2%; 0%], [95% CI: -4%; 1%], respectively) over time (see Table S2). Five early-onset cancer types (C50, C73-C75, C81, C82-C88, C96) exhibited increasing trends among women between 1% and 7%, whereas most early-onset cancers showed neither increasing nor decreasing trends (see Figure S2). For men between 1999 and 2019, the estimation indicated stable incidences for four groups of early-onset cancer (C15-C26, C40-C41, C69-C72, C91-C95) (see Figure S1). However, four cancer types showed slight downward trends between 1% and 3% (C00-C14, C30-C39, C64-C68, C76-C80). All remaining groups of early-onset cancer exhibited increases over time by 1–5% (see Table S2).

Comparing the output of the different statistical methods (see Table S2, S4 and S8) shows that all methods return similar results with only minor differences in the confidence intervals in some few cases. Precisely, the Poisson and negative binomial regression return identical results with only one minor exception in the annual trend estimate of the incidence of melanoma and other malignant neoplasms of skin for women (negative binomial regression: 1.00 [1.00; 1.01] (Table S2) vs. Poisson regression: 1.01 [1.00; 1.01] (Table S4)). Similar findings hold true for joinpoint regression. For both sexes and for the different cancer groups, the estimates deviate by less than 1% (Table S2, S4 and S8). Only one cohort (women, cancer type C96) could not be processed by the Joinpoint Regression Program.

### Mortality

**Fig. 3:**
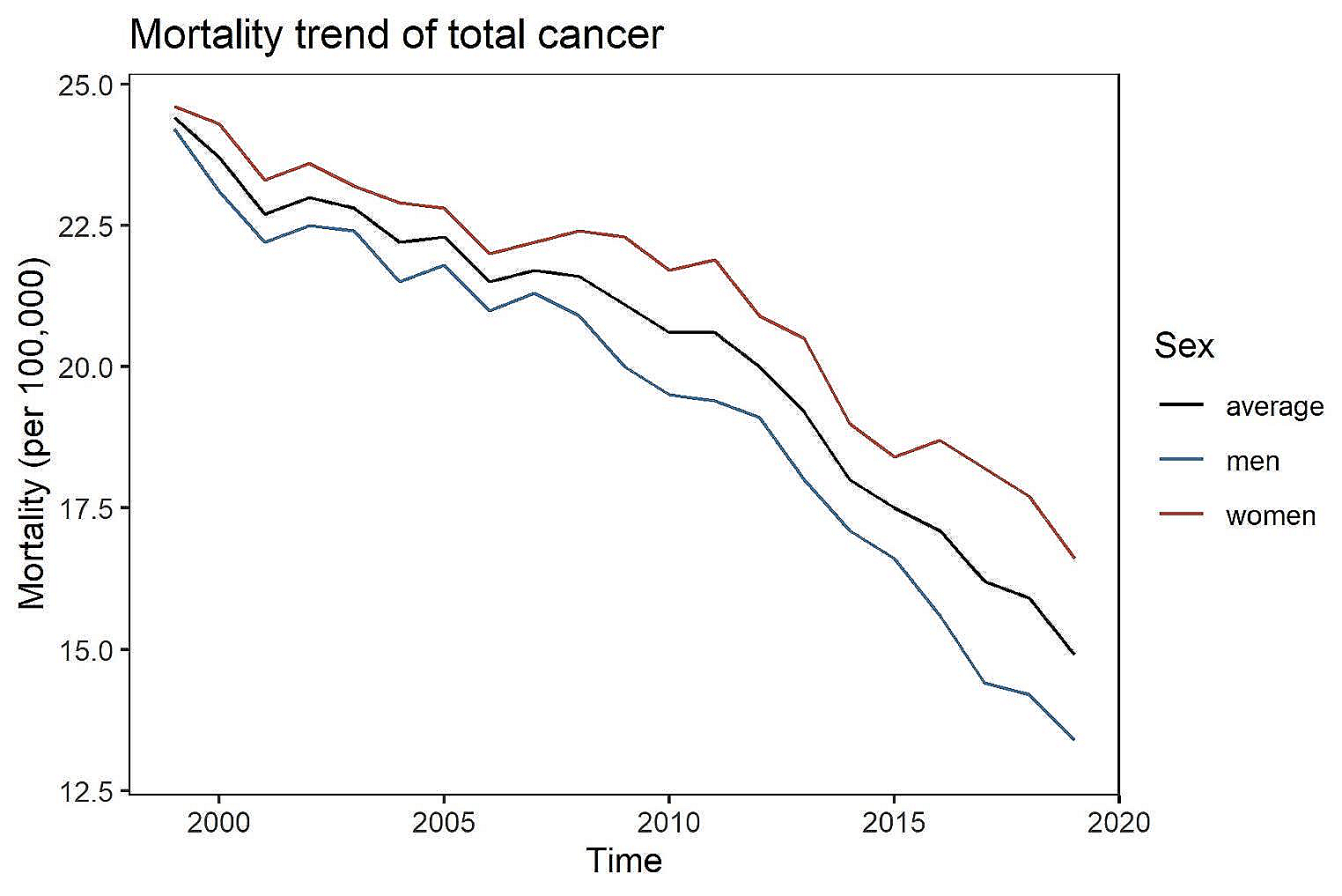
Mortality rate (per 100,000) of all early-onset cancers (C00-C97) combined between 1999 and 2019

A strict downward trend was estimated comparing the mortality of all early-onset cancer types combined in 1999 versus 2019 (see Figs. 2 and 3, Figures S7 and S8). In Germany in 1999, more than 13,000 people aged between 0 and 50 years died from cancer, which was almost twice the number compared to 2019 (approx. 6.800 deaths). With respect to sex, the mortality of all early-onset cancers combined was higher in women compared to men. For all early-onset cancers combined, the regression results indicate a decrease in the mortality by 3% [95% CI: -3%; -2%] among men and 2% [95% CI: -2%; -1%] among women (see Table S3). Only one early-onset cancer type showed an incline over time (4% [95% CI: -20%; 35%] for C96 for women, 1% [95% CI: -4%; 6%] for C73-C75 for men) (Table S3, Figures S3 and S4). Among men, the mortality of four early-onset cancers (C40-C41, C45-C49, C69-C72, C96) showed only slight evidence for a change over time, whereas the mortality of all remaining early-onset cancers showed decreasing trends of up to 6% [95% CI: -8%; -4%]. For women, the results for the cancer types C40-C41, C45-C49 and C73-C75 indicated little evidence for a temporal change (Table S3, Figure S4). The remaining early-onset cancers showed decreasing trends between 1 and 8%, with a maximum decline of 8% [95% CI: -15%; 1%] observed for Hodgkin lymphoma. The mortality was higher with higher age (see Figures S11 and S12). However, higher ages showed the sharpest decline in mortality over time for both men and women.

Detailed findings of our analysis that go beyond the results discussed as well as additional visualisations and detailed results from the overdispersion assessment (Table S7) are provided in the supplementary information.

## Discussion

The aim of this study was to estimate temporal trends in the incidence and mortality of early-onset cancer in Germany between 1999 and 2019. Considering the total early-onset cancer incidence, cancer among younger ages is relatively rare compared to later-onset cancer. Our findings confirm that the incidence is higher as age advances. We observed stable or slightly increasing trends (0% and 1) in the incidence of all early-onset cancers combined (C00-C97) for men and women, respectively, and strict declines in the mortality for men and women (-3% and − 2%).

In Germany, there are no comparable studies on the epidemiology of early-onset cancer. However, the downward trend in the mortality is in line with reported findings on a global scale [1, 4]. Considering the trend in the overall incidence of early-onset cancer in Germany, this is partly conforming with global findings. Between 1990 and until about 2010, the incidence showed an upward trend, which is in line with trend analyses of the global early-onset cancer incidence identified by Lin et al. and Zhao et al. [1, 4]. Contrarily, in Germany since 2010, we noted a decrease in the incidence. This is unlike the findings of Lin et al. [4] and Zhao et al. [1], where the global incidence of early-onset cancer was found to have strictly increased between 1990 and 2019 (by 11% (Lin et al. [4]) and 79.1% (Zhao et al. [1])). With regard to the various, clinically heterogeneous types of early-onset cancer, our results are similar to those of Lin et al. [4] and Zhao et al. [1], seeing that among women, early-onset breast cancer had the highest incidence. Among men, Zhao et al. [1] report a maximum incidence for non-melanoma skin cancer. However, non-melanotic forms of skin cancer (light skin cancer) were not considered in our study due to a lack of data. Our analysis showed that in Germany between 1999 and 2019, malignant neoplasms of male genital organs had the highest incidence. Regarding mortality, early-onset cancer of the eye, brain and other parts of the central nervous system had the highest mortality among women in Germany, which is again in line with Zhao et al. [1] and Lin et al. [4] and their global estimates. For men, the highest mortality in Germany was found for early-onset cancer of digestive organs, while on a global scale, tracheal, bronchus and lung cancer had the highest mortality [1]. However, a recent German study confirmed a relatively high and rising incidence of early-onset colorectal cancer (ICD-10 C18-C20, i.e., part of our classification ICD-10 C15-C26 representing cancer of digestive organs) for the period 1999 to 2018 [2]. Overall, the observed differences between the global findings and our findings for the German population are logical, referring to substantial scientific evidence of considerable variation in the incidence and mortality of early-onset cancer among different areas, ages, countries, genders, and cancer types [1, 4].

As there remains debate about the optimal statistical method for analysing and predicting potential trends over time in cancer-registry data [15, 22], we applied three different statistical methods (negative binomial, Poisson and joinpoint regression). All three methods return comparable outcomes, i.e., negative binomial and Poisson regression return the annual percentage change while the joinpoint regression returns the AAPC. The rationale for applying the three methods is as follows. In epidemiology, Poisson regression is a common methodological choice for assessing trends e.g. in incidence or mortality. As discussed by Alsadhan et al. [22] the second most common statistical method for measuring trends in incidence in cancer registries was Poisson regression. Compared to Poisson regression, negative binomial distribution has one additional parameter and by that, accounts for overdispersion, i.e., it adjusts the variance independently from the mean. With regard to joinpoint regression, we report the AAPC which summarises the average Annual Percent Change (APC) over a period of multiple years in a single number and which is claimed advantageous over the APC [18–20]. First, it does not necessitate linearity of the trend. Second, the AAPC is able to characterize a much longer time series even when the data are sparse. Third, resulting AAPC are discussed as more stable. Fourth, the AAPC is claimed valid even if the joinpoint model indicates changes in trends during the respective time horizon as it is computed as a weighted average of the APCs. Fifth, as single summary measure it increases clarity and better comparability of the results [18–20]. However, modelling trends using piecewise linear segments on a log scale as done by joinpoint regression comes with some limitations (e.g. vs. Poisson or negative binomial regression), such as its inability to deal with missing data and zeros (e.g., in case there are no diseased in a particular cohort, which is not unlikely) [15], its application is much more time consuming [18] and unlike joinpoint regression, Poisson and negative binomial regression additionally return an estimated effect of age on trends in incidence and mortality.

### Causes of the observed cancer burden

The causes for the observed burden of early-onset cancer have not yet been elucidated [1, 2]. Instead, there is growing evidence that the reasons and risk factors are multifactorial with differences between the specific cancer types, sex and age, demographics, society, economy, and lifestyle [1, 2, 4, 5]. For instance with regard to sex and on overall level, i.e. for all cancer types combined, we found an increase in the incidence among women and a stable incidence among men. The ZfKD argues that this difference is mainly due to sex-specific trends in respiratory and intrathoracic organs (C30-39), mostly lung cancer (C34), and other cancers promoted by cigarette smoking, which have been decreasing for men (by 3% in our study) [23]. Furthermore, changes in the environment (e.g., higher pollution rates) combined with the Western lifestyle and diet (e.g., high consumption of red meat, animal fats, refined carbohydrates, alcohol) and the resulting increases in obesity and physical inactivity are likely to have affected (and potentially increased) the incidence of early-onset cancer [1, 4, 23, 24]. Moreover, exposure to the mentioned risk factors already in early life or young adulthood might be another reason for the rising incidence of early-onset cancer. The exposure to such risk factors has likely caused the increasing incidence for both sexes (e.g., breast cancer (C50), thyroid and endocrine glands (C73-C75), Hodgkin Lymphoma (C81), Non-Hodgkin Lymphoma (C82-88), multiple independent sites (C96) for men and women; and increases in cancer in melanoma (C43), soft tissue (C45-49), male genitals (C60-63), myeloma (C90) among men), at least to a certain extent. Another reason for increasing incidence for some cancer types may be the promotion of cancer screening strategies, such as the worldwide extensive application and promotion of mammography screening between 2005 and 2015 [1, 4]. For instance, after the introduction of mammography screening in Germany in 2009, there is substantial evidence for an increase in the breast cancer (C50) incidence among the screened age-groups [25]. This may explain part of the increase in the incidence of early-onset breast cancer (C50) found in our analysis.

However, although there is an increasing uptake of screening and early detection activities, only few of the screening strategies for detectable cancers aim at individuals who are aged younger than 50 years [26]. For instance, only men aged 45 and over are eligible for prostate cancer screening, colonoscopy is available for women and men aged 55 years and over and mammography is offered for women aged between 50 and 70 years [26, 27]. Presumably, this explains a major part of the stable incidence rates of prostate cancer (C61) and colorectal cancer (C18) we observed in our analysis. Further, participation in cancer screening in Germany is voluntary [26, 27]. With regard to mammography, the attendance probability of the German population in 2014 was about 50%, i.e., only half of the eligible German population [25]. In addition, some examinations and tests (individual health services, in German: individuelle Gesundheitsleistungen, abbreviated to “IGeL”) are not part of the statutory early detection program and costs have to be paid for privately [27]. These include for instance ultrasound examination of the ovaries and prostate or the inspection of blood or other bodily fluids in the laboratory. It is not inconceivable that particularly young adults who do not represent major risk groups are less willing to pay for such screening possibilities.

Similarly as for the increasing incidence for some cancer types, the decline in cancer incidence (observed among women for female genital organs (C51-C58) and unspecified sites of cancer (C76-C89); among men for lip, oral cavity and pharynx cancer (C00-C14), respiratory organs (C30-C39), urinary tract (C64-C68) and unspecified sites (C76-C80)) might be caused by a multitude of impacting factors. The strength and prevalence of risk factors as well as advancements in cancer screening implemented before or during the study period, it is important to consider latency effects, i.e. the temporal delay of a potential reaction to changes, which could impact the observed incidence trends. For instance, tobacco has a long latency (about 10 years) for its effect on lung carcinoma, whereas diet and nutritional factors, treatment and medication as well as screening are said to have shorter latencies (about 5 years) [24]. It is thus important to consider the time it may take to impact (both, reduce or increase) cancer incidence rates subsequent to a change. Particularly with regard to smoking, it is well known that tobacco increases the risk of cancer in respiratory and intrathoracic organs, but also at many sites other than the lung, the most common being the oral cavity, pharynx, larynx, oesophagus, and urinary bladder [23, 24]. In Germany, smoking bans have been in effect in all federal states since July 1, 2008. Subsequent reductions in tobacco consumption and (active and passive) smoking are thus the most likely cause of the observed decline in lung cancer and other tobacco-related cancers (e.g., respiratory and intrathoracic organs (C30-39), urinary tract (C64-68), unspecified sites (C76-80)) in our analysis.

Comparing the development of early-onset to late-onset cancer showed that on overall level, i.e., all cancer types combined (C00-C96), there is a substantial difference in the absolute level of the incidence. The incidence of late-onset cancer is almost four times higher compared to the incidence of early-onset cancer. However, the temporal trend developed similarly between 1999 and 2019. This might indicate that changes in risk factors, screening, cancer management or similar might have had larger impact on cancer incidence (for both, early- and late-onset) than different underlying biological mechanisms of early- vs. late-onset cancer. However, this inference should be made with caution. More detailed future research is required to make this statement with certainty.

As for the incidence, a number of factors may be responsible for the decrease in cancer mortality. Firstly, screening and the consequent earlier detection of symptomatic disease may have led to subsequent reductions in mortality. For instance, the efficacy of early breast cancer detection through mammography and clinical breast examination has reduced breast cancer mortality by up to 25% between 2009 and 2016 [25]. Secondly, treatment and cancer management (e.g., improved surgical technique, standardization of preoperative and postoperative care, medication) today are better, more effective and more easily available in Germany than they were three decades ago.

### Implications for cancer management

The incidence and mortality were largely variant with regard to the different cancer types, sex and age. As a consequence, high focus should be placed on local characteristics and prevailing risk factors when formulating cancer prevention and treatment strategies. Further, although it seems that the incidence of early-onset cancer in Germany has decreased over the last decade, it should be noted that this is only the case for some cancer types. Consequently, future emphasis should be placed on cancer prevention and cancer health education, on increasing public awareness of the multitude of potential risk factors, and on developing effective and targeted screening strategies for the various cancer types. However, the mechanisms of several cancer types are not yet fully known or the known causes cannot be influenced. Therefore, efforts should be placed on future research of prevention opportunities, to increase the estimated 40% of all cancer cases worldwide which could be avoided through preventive measures [23]. For instance, primary focus should be placed on early prevention practices through promoting balanced and healthy nutrition, regular exercising, maintaining a healthy body weight, timely childbirth, and breastfeeding. There is substantial scientific evidence that for instance, consuming about 5 servings of fruits and vegetables per day inhibits the risk of colorectal carcinoma. Other changes in diet that should be promoted particularly among the youth are less alcohol consumption, reduced consumption of smoked, red and cured meats which are all known and modifiable risk factors. Furthermore, the widespread use of antibiotics can lead to changes in gut flora and affect the gastrointestinal microbiome. As such, these drugs function as cancer risk factor. It may be valuable to consider a stricter prescription process of pain killers and antibiotics, and making such drugs less easily available (particularly for children and young adults). Another modifiable risk factor are chronic infections. In Germany, about 4% of all incident cases per year are attributable to infections [23]. Vaccinations could aid in the first place to avert chronic conditions and by that, any subsequent cancer risk. This has been shown for the hepatitis B vaccination, as well as for the HPV vaccination. It may therefore be of value to promote vaccinations among the youth and to create incentives for being vaccinated early and regularly (in case repeated vaccination is required to maintain protection). Further efforts should be placed on early detection of cancer and making screening available for children, teenagers and young adults, too. For instance, the introduction of mammography screening in Germany in 2009 led to a substantial increase in the breast cancer (C50) incidence. On the grounds of these findings, it seems recommendable to establish early and regular routine screening activities for women and men at younger ages (i.e., far below the ages of 50 years), too. In addition, it might be valuable to extend cancer databases and to more extensively collect information on screening methods and their application. For example, tumour registries could be complemented by adding genetic and preventive information as suggested by Schmutzler et al. [28]. In doing so, the effectiveness of preventive measures based on validated risk factors and screening activities could be better evaluated in general as well as potentially, such information may provide evidence that allows for a transition from age- to risk-adjusted screening. Associations of smoking and tobacco consumptions with higher cancer risk have also been shown in numerous observational studies and have thus been identified as leading risk factors for cancer in respiratory and intrathoracic organs, but also at many sites other than the lung, such as the oral cavity, pharynx, larynx, oesophagus, and urinary bladder [23, 24]. Good efforts in reducing the tobacco consumption such as though the smoking ban since 2008 should be maintained, if not even extended. The passing of the cannabis act (Cannabisgesetz, CanG) in Germany in April 2024 legalises private cultivation of cannabis by adults for their own consumption and allows smoking joints legally. It is well known that cannabis may relieve many of the symptoms that can occur among cancer patients, including sleep problems, nausea, loss of appetite, weakness and pain [29, 30]. However, studies found an association of cannabis and lung cancer risk as well as a weakening of the body’s immune system, which in turn increases the susceptibility to infectious diseases and risk of cancer [29–31]. It may be wise to identify major consumer profiles (i.e., potential risk groups), to closely monitor the future cannabis consumption and to observe its consequences [31].

With regards to trends in mortality in Germany, the generally low and decreasing rates may be due to sufficient local medical resources, as well as effective prevention and control practices. Further, the observed reduction of cancer mortality may imply that earlier efforts in medical development and treatment activities have achieved good results in cancer management. Furthermore, it must be noted that despite decreasing incidence, a disease’s prevalence may increase, for instance, due to ameliorated medical care, improvements in disease treatment and/or a reduction in disease-related mortality. Our results should therefore not be mistaken as an indication of decreasing total cancer burden among young adults and the associated demand on healthcare resources. Consequently, it is key to maintain these efforts and to further invest in averting the burden of early-onset cancer in terms of cancer being the main disease hindering life expectancy.

### Strength and weaknesses

A main strength of our study is that it is based on nationwide registry data from the German population [9]. By contrast, one of the major limitations of previous is that the accuracy of GBD data was compromised by the quality, availability and representativeness of cancer registry data for the different countries. Hence, using data obtained from the Centre for Cancer Registry Data (Zentrum für Krebsregisterdaten, ZfKD) our study overcomes this major limitation of previous global cancer studies. The described data enabled us to draw a relatively comprehensive and unbiased picture of temporal trends in the incidence and mortality of diagnosed early-onset cancer in Germany for a long observation period of two decades. Another advantage is that our analysis differentiates by sex and age, as well as by cancer type. Knowing that cancer combines several heterogeneous subtypes in terms of different aetiologies, physiologies, risk factors, and treatments, our findings quantify and clarify the burden of the specific cancer types for each sex. Lastly, we applied three different statistical models to estimate the potential trends. The results from all methods are consistent and validate the respective findings.

However, the present study is subject to some limitations that are mostly related to our input data. Firstly, with the passing of the Federal Cancer Registry Act (BKRG) in Germany in 1995, all German states are obligated to establish epidemiological cancer registries and pass their data to a national cancer registry. The law has greatly advanced cancer registration such that nowadays, the cancer registry data of the ZfKD has a sufficiently high coverage of over 90%. However, it does not (yet) achieve 100% completeness (see Table S6) and thus leaves room for some uncertainties in the representativeness of our estimates [9, 13]. Further, due to potential differences in the quality of the cancer registry data in the different federal states, under-recording or underdiagnosis may occur which may result in underestimation [32]. Secondly, the data does not allow for precise reflection on cancer survivors, i.e., people who are considered cured when having lived an average of five years after successful cancer therapy without recurrence [33]. We are thus unable to make any statements concerning the number of unrecorded cases, i.e., undiagnosed or not yet detected cancer. Consequently, we are only making statements about the rates and trends of diagnosed cancer. The total incidence of diagnosed and undiagnosed early-onset cancer is likely higher than our estimate since people with undiagnosed cancer are not considered in our estimation. However, this is only relevant and may impact the estimated temporal trend if the proportion of diagnosed compared to undiagnosed cancer cases has changed between 1999 and 2019. Thirdly, our input data was aggregated on population level and was differentiated only with regard to age and sex. Therefore, due to lacking information, we stratified our analysis accounting for sex and age only. However, other risk factors or covariates may be worth assessing in this context. Fourth, we aligned with the German cancer registry and the ICD classification with regard to the grouping of the different cancer types [5]. Knowing that cancer is a group of various subtypes which differ greatly by type and among different patients, it may be of interest to further split the groups and consider each type (C00 to C97) and its subtypes separately. For example, esophageal adenocarcinoma is different from esophageal squamous carcinoma as well as microsatellite instability-high colorectal cancer is different from non-MSI colorectal cancer. It may be possible that the grouping of different cancer types may mask significant increasing or decreasing trends in each cancer entity. Consequently, future studies that investigate the incidence and mortality of each early-onset cancer are warranted. This may be helpful to counteract the individual types of cancer more effectively and in a more tailored manner.

## Conclusion

We advocate to closely monitor early-onset cancer in Germany to observe whether the decreasing trends continue or whether the increasing trends reverse. It may seem that rates of some cancers are being brought under control through the decreasing prevalence of known risk factors, early detection, and improved treatment. Nonetheless, it is important to consider that, despite decreasing incidence, the prevalence of a disease can rise (e.g. due to improvements in medical care, better disease treatment or reduced mortality). Consequently, our findings do not necessarily indicate a decrease in the total cancer disease burden among young adults and the associated demand on healthcare resources. Overall, we suggest that effective and locally tailored cancer prevention and control measures are essential in further reducing and controlling the cancer burden in the younger German population.

### Electronic supplementary material

Below is the link to the electronic supplementary material.

Supplementary Material 1

## Data Availability

All input data is freely available at the data base maintained by the Centre for Cancer Registry Data (Zentrum für Krebsregisterdaten, ZfKD) of the Robert Koch Institute (RKI). The source code of our analysis is publicly available on Zenodo [1].
