# Correction: Trends in incidence and mortality of early-onset cancer in Germany between 1999 and 2019

**DOI:** 10.1007/s10654-024-01143-3

**Published:** 2024-08-11

**Authors:** Dina Voeltz, Kira Baginski, Claudia Hornberg, Annika Hoyer

**Affiliations:** 1 Biostatistics and Medical Biometry, Medical School OWL, Bielefeld University, Bielefeld, Germany; 2 Department of Statistics, Ludwig-Maximilians-University Munich, Munich, Germany; 3 Department of Environmental Health Sciences, Medical School OWL, Bielefeld University, Bielefeld, Germany

**Correction: European Journal of Epidemiology**

10.1007/s10654-024-01134-4.

In the sentence beginning ‘In Germany in….’ in the results section, the text ‘In Germany in 2019, the age-standardised incidence of all early-onset cancer types combined was 375.8 per 100,000, a 2% decrease compared to 1999 (384.6 per 100,000) (see Fig. 1).’ should have read ‘In Germany in 2019, the age-standardised incidence of all early-onset cancer types combined was 95.2 per 100,000, a 3% increase compared to 1999 (92.0 per 100,000) (see Fig. 1).’

In this article the wrong figure appeared as Figs. 1 & 2. The original Fig. 1 erroneously visualizes the age-standardized incidence for the overall German population including all ages from 0 to 100, i.e. it is not restricted to early-onset cancer. The corrected version of Fig. 1 is limited to the incidence of early-onset cancer (0 to 50 years). In the original Fig. 2, the x-axes of the panels related to the incidence (left part of the figure) are not correct. The revised version indicates the correct scale. We further added a detailed explanation of category C96. The figures should have appeared as shown below.

The authors regret these errors.

**Fig. 1:**
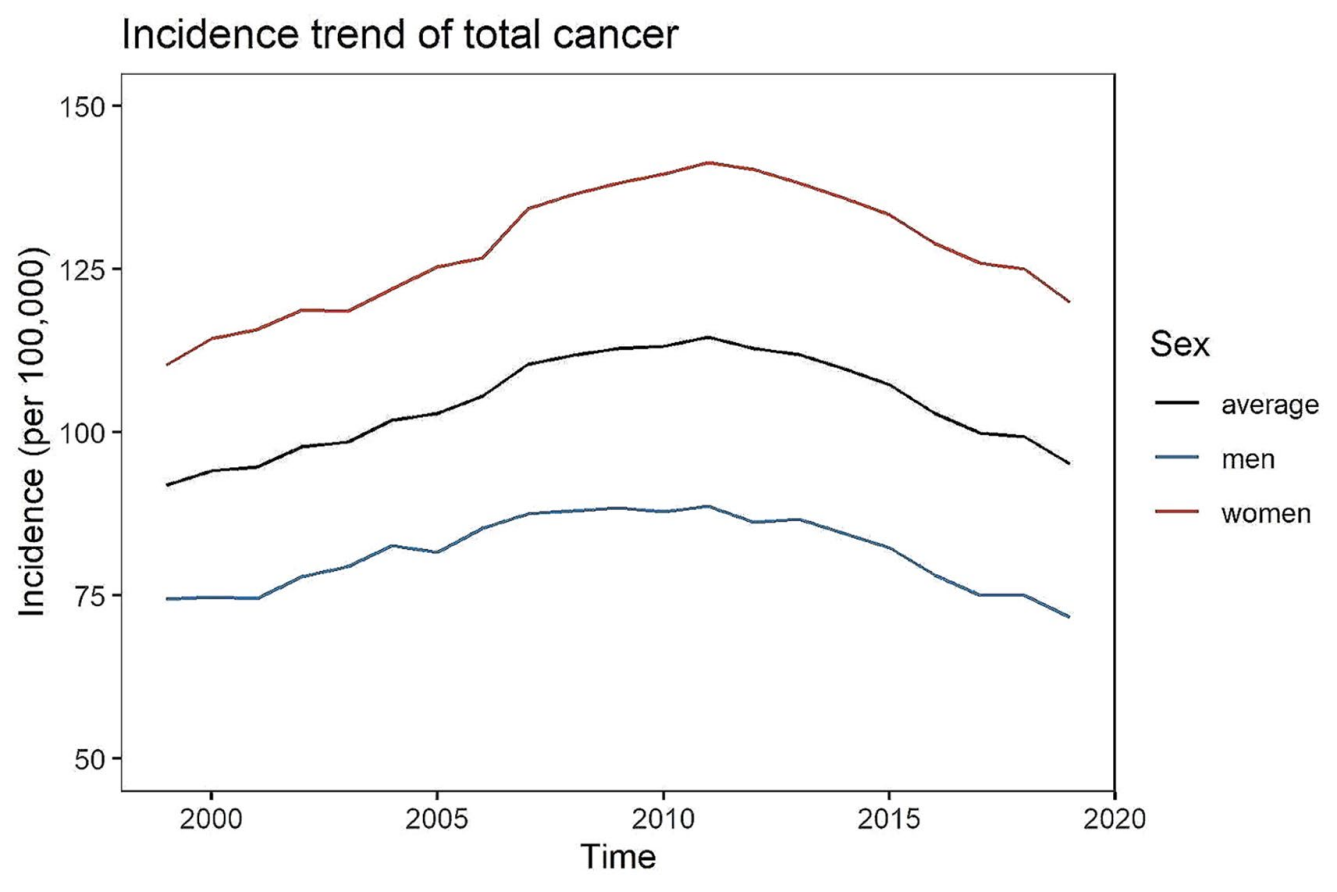
Age-standardized incidence rate (per 100,000) of all early-onset cancers (C00-C97) combined between 1999 and 2019

**Fig. 2:**
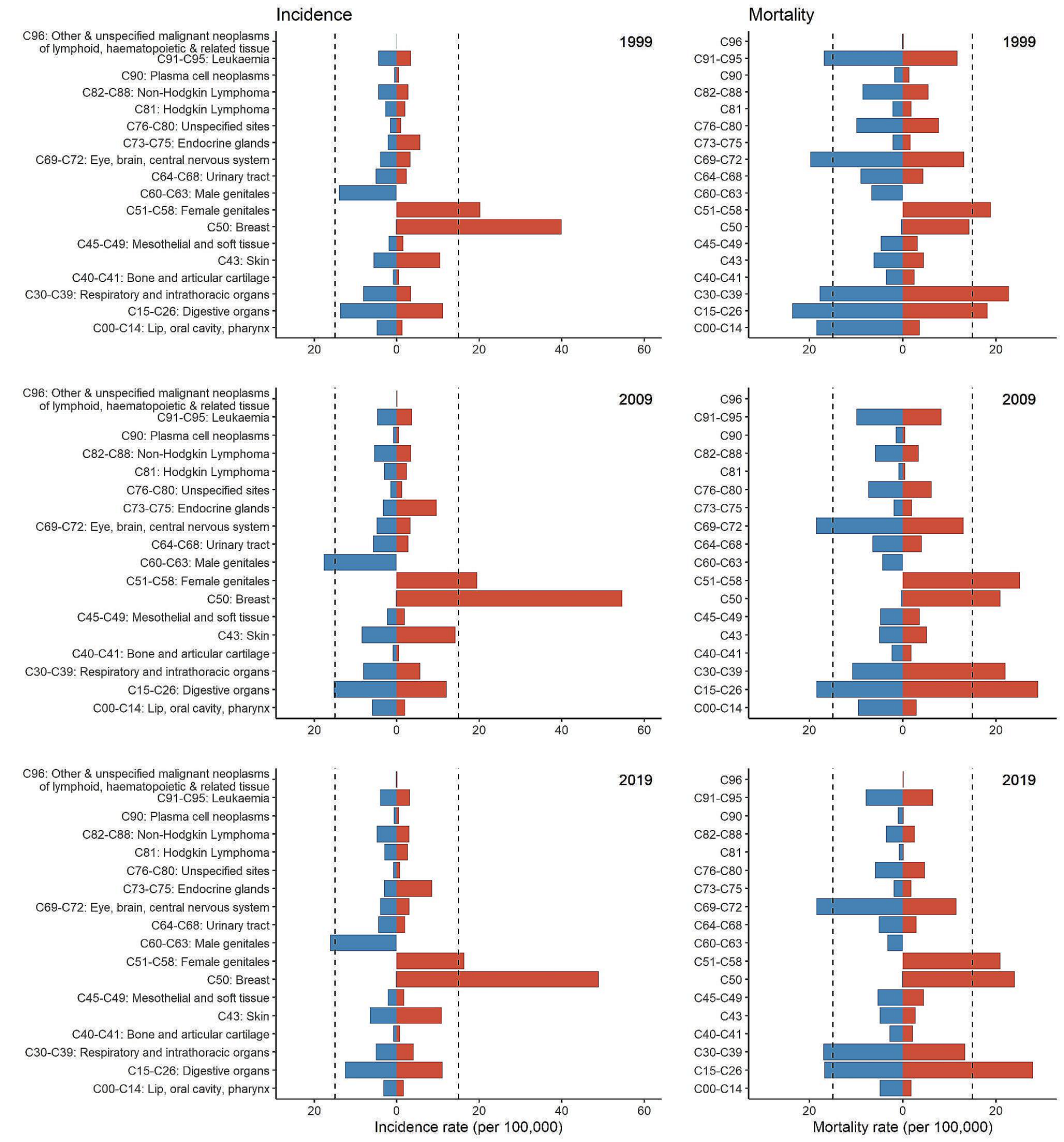
Incidence and mortality rate in 1999, 2009 and 2019 by sex and cancer type

