# Supplementary material for: Trends in incidence and mortality of early-onset cancer in Germany between 1999 and 2019
Source: Eur J Epidemiol. 2024 May 31;39(7):827–37. doi: 10.1007/s10654-024-01134-4 (PMC11343808; doi:10.1007/s10654-024-01134-4)
Supplement: Supplementary file 1 — Supplementary Material 1 [file 10654_2024_1134_MOESM1_ESM.docx]

# Supplementary Information to

# Trends in the incidence and mortality of early-onset cancer in Germany between 1999 and 2019

## Definition and Grouping of Cancer Types

To group the 19 groups of cancer (18 different groups + 1 total cancer group), we used the following system based on International Classification of Diseases (ICD) coding. We defined total cancer as all malignant neoplasms, including lymphomas and leukaemia (ICD-10 C00-C97, excl. C44). Non-melanotic forms of skin cancer (light skin cancer), i.e., C44, were not considered. Despite their frequency, the contribution of these cancer types to total cancer mortality is negligible [1, 2].

Table S1: Grouping of cancer types

| **No.** | **ICD-10** | **Description** |
| --- | --- | --- |
| 1 | C00 – C97 (excl. C44) | Total cancer |
| 2 | C00 – C14 | Malignant neoplasms of lip, oral cavity and pharynx |
| 3 | C15 – C26 | Malignant neoplasms of digestive organs |
| 4 | C30 – C39 | Malignant neoplasms of respiratory and intrathoracic organs |
| 5 | C40 – C41 | Malignant neoplasms of bone and articular cartilage |
| 6 | C43 | Melanoma and other malignant neoplasms of skin |
| 7 | C45 – C49 | Malignant neoplasms of mesothelial and soft tissue |
| 8 | C50 | Malignant neoplasm of breast |
| 9 | C51 – C58 | Malignant neoplasms of female genital organs |
| 10 | C60 – C63 | Malignant neoplasms of male genital organs |
| 11 | C64 – C68 | Malignant neoplasms of urinary tract |
| 12 | C69 – C72 | Malignant neoplasms of eye, brain and other parts of central nervous system |
| 13 | C73 – C75 | Malignant neoplasms of thyroid and other endocrine glands |
| 14 | C76 – C80 | Malignant neoplasms of ill-defined, secondary and unspecified sites |
| 15 | C81 | Hodgkin Lymphoma |
| 16 | C82 – C88 | Non-Hodgkin-Lymphoma |
| 17 | C90 | Multiple myeloma and malignant plasma cell neoplasms |
| 18 | C91 – C95 | Leukaemia |
| 19 | C96 | Malignant neoplasms of independent (primary) multiple sites |

## Negative Binomial Regression Output

Table S2: Results from the negative binomial regression estimating trends in cancer incidence

|  |  | **Men** | | | | **Women** | | | |
| --- | --- | --- | --- | --- | --- | --- | --- | --- | --- |
| **ICD** |  | **RR** | **[95%-CI]** | | **p-value** | **RR** | **[95%-CI]** | | **p-value** |
| C00-C97 | year | 1.00 | 1.00 | 1.01 | 0.45 | 1.01 | 1.00 | 1.01 | 0.00 |
|  | age | 1.07 | 1.07 | 1.07 | 0.00 | 1.09 | 1.09 | 1.10 | 0.00 |
| C00-C14 | year | 0.98 | 0.97 | 0.99 | 0.00 | 1.00 | 0.99 | 1.02 | 0.65 |
|  | age | 1.18 | 1.16 | 1.19 | 0.00 | 1.12 | 1.11 | 1.14 | 0.00 |
| C15-C26 | year | 1.00 | 0.99 | 1.00 | 0.22 | 1.00 | 0.99 | 1.01 | 0.65 |
|  | age | 1.15 | 1.14 | 1.15 | 0.00 | 1.12 | 1.12 | 1.13 | 0.00 |
| C30-C39 | year | 0.97 | 0.96 | 0.98 | 0.00 | 1.00 | 0.99 | 1.01 | 0.97 |
|  | age | 1.18 | 1.17 | 1.20 | 0.00 | 1.18 | 1.17 | 1.20 | 0.00 |
| C40-C41 | year | 1.00 | 0.98 | 1.03 | 0.70 | 1.00 | 0.97 | 1.03 | 0.99 |
|  | age | 1.00 | 0.99 | 1.01 | 0.84 | 1.00 | 0.99 | 1.01 | 0.90 |
| C43 | year | 1.02 | 1.01 | 1.03 | 0.00 | 1.00 | 0.99 | 1.01 | 0.96 |
|  | age | 1.09 | 1.08 | 1.10 | 0.00 | 1.10 | 1.09 | 1.11 | 0.00 |
| C45-C49 | year | 1.01 | 0.99 | 1.02 | 0.41 | 1.00 | 0.99 | 1.02 | 0.74 |
|  | age | 1.03 | 1.02 | 1.04 | 0.00 | 1.02 | 1.02 | 1.03 | 0.00 |
| C50 | year | 1.02 | 0.96 | 1.08 | 0.56 | 1.01 | 1.00 | 1.02 | 0.01 |
|  | age | 1.17 | 1.11 | 1.24 | 0.00 | 1.19 | 1.18 | 1.19 | 0.00 |
| C51-C58 | year |  |  |  |  | 0.99 | 0.98 | 1.00 | 0.02 |
|  | age |  |  |  |  | 1.11 | 1.11 | 1.12 | 0.00 |
| C60-C63 | year | 1.01 | 0.99 | 1.02 | 0.41 |  |  |  |  |
|  | age | 1.09 | 1.09 | 1.10 | 0.00 |  |  |  |  |
| C64-C68 | year | 0.99 | 0.98 | 1.00 | 0.10 | 1.00 | 0.98 | 1.01 | 0.59 |
|  | age | 1.12 | 1.11 | 1.13 | 0.00 | 1.07 | 1.06 | 1.08 | 0.00 |
| C69-C72 | year | 1.00 | 0.99 | 1.01 | 0.63 | 1.00 | 0.99 | 1.02 | 0.51 |
|  | age | 1.03 | 1.02 | 1.03 | 0.00 | 1.02 | 1.02 | 1.03 | 0.00 |
| C73-C75 | year | 1.02 | 1.01 | 1.04 | 0.00 | 1.03 | 1.02 | 1.03 | 0.00 |
|  | age | 1.05 | 1.05 | 1.06 | 0.00 | 1.06 | 1.06 | 1.07 | 0.00 |
| C76-C80 | year | 0.97 | 0.95 | 0.99 | 0.01 | 0.99 | 0.96 | 1.01 | 0.26 |
|  | age | 1.11 | 1.10 | 1.13 | 0.00 | 1.12 | 1.10 | 1.13 | 0.00 |
| C81 | year | 1.01 | 1.00 | 1.02 | 0.16 | 1.01 | 1.00 | 1.03 | 0.11 |
|  | age | 1.02 | 1.02 | 1.03 | 0.00 | 1.01 | 1.01 | 1.02 | 0.00 |
| C82-C88 | year | 1.01 | 1.00 | 1.02 | 0.11 | 1.01 | 0.99 | 1.02 | 0.39 |
|  | age | 1.06 | 1.05 | 1.06 | 0.00 | 1.07 | 1.06 | 1.08 | 0.00 |
| C90 | year | 1.01 | 0.98 | 1.04 | 0.57 | 1.00 | 0.97 | 1.04 | 0.79 |
|  | age | 1.19 | 1.15 | 1.23 | 0.00 | 1.19 | 1.15 | 1.23 | 0.00 |
| C91-C95 | year | 1.00 | 0.99 | 1.01 | 0.99 | 1.00 | 0.99 | 1.01 | 0.78 |
|  | age | 1.00 | 1.00 | 1.01 | 0.28 | 1.00 | 0.99 | 1.00 | 0.67 |
| C96 | year | 1.05 | 0.99 | 1.12 | 0.10 | 1.07 | 0.98 | 1.17 | 0.15 |
|  | age | 1.04 | 1.01 | 1.07 | 0.01 | 1.05 | 1.00 | 1.09 | 0.04 |
| **RR = relative risk; 95%-CI = 95% confidence interval* | | | | | | | | | |

Table S3: Results from the negative binomial regression estimating trends in cancer mortality

|  |  | **Men** |  |  |  | **Women** |  |  |  |
| --- | --- | --- | --- | --- | --- | --- | --- | --- | --- |
| **ICD** |  | **RR** | **[95%-CI]** | | **p-value** | **RR** | **[95%-CI]** | | **p-value** |
| C00-C97 | year | 0.97 | 0.97 | 0.98 | 0.00 | 0.98 | 0.98 | 0.99 | 0.00 |
|  | age | 1.10 | 1.09 | 1.11 | 0.00 | 1.12 | 1.12 | 1.12 | 0.00 |
| C00-C14 | year | 0.94 | 0.92 | 0.96 | 0.00 | 0.96 | 0.92 | 1.01 | 0.08 |
|  | age | 1.24 | 1.21 | 1.28 | 0.00 | 1.18 | 1.12 | 1.23 | 0.00 |
| C15-C26 | year | 0.98 | 0.97 | 0.99 | 0.00 | 0.99 | 0.97 | 1.00 | 0.03 |
|  | age | 1.18 | 1.16 | 1.19 | 0.00 | 1.16 | 1.14 | 1.17 | 0.00 |
| C30-C39 | year | 0.95 | 0.94 | 0.96 | 0.00 | 0.97 | 0.96 | 0.99 | 0.00 |
|  | age | 1.24 | 1.22 | 1.26 | 0.00 | 1.22 | 1.19 | 1.24 | 0.00 |
| C40-C41 | year | 1.00 | 0.96 | 1.05 | 0.85 | 1.00 | 0.95 | 1.05 | 0.95 |
|  | age | 1.01 | 0.99 | 1.03 | 0.30 | 1.01 | 0.99 | 1.03 | 0.43 |
| C43 | year | 0.99 | 0.96 | 1.02 | 0.37 | 0.99 | 0.96 | 1.02 | 0.57 |
|  | age | 1.12 | 1.10 | 1.15 | 0.00 | 1.12 | 1.09 | 1.15 | 0.00 |
| C45-C49 | year | 1.00 | 0.97 | 1.03 | 0.95 | 1.00 | 0.97 | 1.04 | 0.82 |
|  | age | 1.05 | 1.03 | 1.06 | 0.00 | 1.05 | 1.03 | 1.07 | 0.00 |
| C50 | year | 0.96 | 0.84 | 1.09 | 0.53 | 0.98 | 0.97 | 0.99 | 0.00 |
|  | age | 1.14 | 1.03 | 1.27 | 0.02 | 1.17 | 1.16 | 1.18 | 0.00 |
| C51-C58 | year |  |  |  |  | 0.98 | 0.97 | 0.99 | 0.00 |
|  | age |  |  |  |  | 1.15 | 1.13 | 1.16 | 0.00 |
| C60-C63 | year | 0.97 | 0.94 | 1.01 | 0.12 |  |  |  |  |
|  | age | 1.09 | 1.06 | 1.11 | 0.00 |  |  |  |  |
| C64-C68 | year | 0.97 | 0.95 | 1.00 | 0.05 | 0.99 | 0.96 | 1.03 | 0.67 |
|  | age | 1.16 | 1.13 | 1.20 | 0.00 | 1.11 | 1.08 | 1.14 | 0.00 |
| C69-C72 | year | 1.00 | 0.98 | 1.01 | 0.68 | 0.99 | 0.97 | 1.01 | 0.28 |
|  | age | 1.05 | 1.05 | 1.06 | 0.00 | 1.05 | 1.04 | 1.06 | 0.00 |
| C73-C75 | year | 1.01 | 0.96 | 1.06 | 0.80 | 1.00 | 0.95 | 1.05 | 0.98 |
|  | age | 1.02 | 1.00 | 1.04 | 0.13 | 1.02 | 1.00 | 1.05 | 0.05 |
| C76-C80 | year | 0.97 | 0.95 | 1.00 | 0.04 | 0.98 | 0.95 | 1.01 | 0.21 |
|  | age | 1.11 | 1.09 | 1.13 | 0.00 | 1.12 | 1.09 | 1.14 | 0.00 |
| C81 | year | 0.95 | 0.89 | 1.02 | 0.18 | 0.92 | 0.85 | 1.01 | 0.07 |
|  | age | 1.07 | 1.03 | 1.11 | 0.00 | 1.05 | 1.01 | 1.09 | 0.02 |
| C82-C88 | year | 0.97 | 0.94 | 1.00 | 0.03 | 0.96 | 0.93 | 1.00 | 0.04 |
|  | age | 1.08 | 1.06 | 1.10 | 0.00 | 1.07 | 1.05 | 1.10 | 0.00 |
| C90 | year | 0.98 | 0.92 | 1.04 | 0.43 | 0.96 | 0.89 | 1.04 | 0.28 |
|  | age | 1.24 | 1.14 | 1.34 | 0.00 | 1.22 | 1.11 | 1.34 | 0.00 |
| C91-C95 | year | 0.97 | 0.95 | 0.99 | 0.01 | 0.97 | 0.95 | 1.00 | 0.03 |
|  | age | 1.03 | 1.02 | 1.04 | 0.00 | 1.03 | 1.02 | 1.04 | 0.00 |
| C96 | year | 1.00 | 0.77 | 1.29 | 0.99 | 1.04 | 0.80 | 1.35 | 0.78 |
|  | age | 1.04 | 0.92 | 1.16 | 0.55 | 1.00 | 0.89 | 1.11 | 0.93 |
| **RR = relative risk; 95%-CI = 95% confidence interval* | | | | | | | | | |

## Possion Regression Output

Table S4: Results from the Poisson regression estimating trends in cancer incidence

|  | |  | **Men** | | | | | **Women** | | | |
| --- | --- | --- | --- | --- | --- | --- | --- | --- | --- | --- | --- |
| **ICD** |  | | **RR** | **[95%-CI]** | | **p-value** | **RR** | | **[95%-CI]** | | **p-value** |
| C00-C97 | year | | 1.00 | 1.00 | 1.00 | 0.85 | 1.01 | | 1.00 | 1.01 | 0.00 |
| C00-C97 | age | | 1.07 | 1.07 | 1.08 | 0.00 | 1.09 | | 1.09 | 1.10 | 0.00 |
| C00-C14 | year | | 0.98 | 0.97 | 0.99 | 0.00 | 1.00 | | 0.99 | 1.02 | 0.65 |
| C00-C14 | age | | 1.18 | 1.16 | 1.19 | 0.00 | 1.12 | | 1.11 | 1.14 | 0.00 |
| C15-C26 | year | | 1.00 | 0.99 | 1.00 | 0.22 | 1.00 | | 0.99 | 1.01 | 0.65 |
| C15-C26 | age | | 1.15 | 1.14 | 1.15 | 0.00 | 1.12 | | 1.12 | 1.13 | 0.00 |
| C30-C39 | year | | 0.97 | 0.96 | 0.98 | 0.00 | 1.00 | | 0.99 | 1.01 | 0.97 |
| C30-C39 | age | | 1.18 | 1.17 | 1.20 | 0.00 | 1.18 | | 1.17 | 1.2 | 0.00 |
| C40-C41 | year | | 1.00 | 0.98 | 1.03 | 0.70 | 1.00 | | 0.97 | 1.03 | 0.99 |
| C40-C41 | age | | 1.00 | 0.99 | 1.01 | 0.84 | 1.00 | | 0.99 | 1.01 | 0.90 |
| C43 | year | | 1.02 | 1.01 | 1.03 | 0.00 | 1.01 | | 1.00 | 1.01 | 0.05 |
| C43 | age | | 1.09 | 1.08 | 1.10 | 0.00 | 1.08 | | 1.07 | 1.08 | 0.00 |
| C45-C49 | year | | 1.01 | 0.99 | 1.02 | 0.41 | 1.00 | | 0.99 | 1.02 | 0.74 |
| C45-C49 | age | | 1.03 | 1.02 | 1.04 | 0.00 | 1.02 | | 1.02 | 1.03 | 0.00 |
| C50 | year | | 1.02 | 0.96 | 1.08 | 0.56 | 1.01 | | 1.01 | 1.01 | 0.00 |
| C50 | age | | 1.17 | 1.11 | 1.24 | 0.00 | 1.15 | | 1.15 | 1.16 | 0.00 |
| C51-C58 | year | |  |  |  |  | 0.99 | | 0.99 | 1.00 | 0.00 |
| C51-C58 | age | |  |  |  |  | 1.11 | | 1.1 | 1.11 | 0.00 |
| C60-C63 | year | | 1.01 | 1.00 | 1.01 | 0.02 |  | |  |  |  |
| C60-C63 | age | | 1.06 | 1.06 | 1.07 | 0.00 |  | |  |  |  |
| C64-C68 | year | | 0.99 | 0.98 | 1.00 | 0.10 | 1.00 | | 0.98 | 1.01 | 0.59 |
| C64-C68 | age | | 1.12 | 1.11 | 1.13 | 0.00 | 1.07 | | 1.06 | 1.08 | 0.00 |
| C69-C72 | year | | 1.00 | 0.99 | 1.01 | 0.63 | 1.00 | | 0.99 | 1.02 | 0.51 |
| C69-C72 | age | | 1.03 | 1.02 | 1.03 | 0.00 | 1.02 | | 1.02 | 1.03 | 0.00 |
| C73-C75 | year | | 1.02 | 1.01 | 1.04 | 0.00 | 1.03 | | 1.02 | 1.03 | 0.00 |
| C73-C75 | age | | 1.05 | 1.05 | 1.06 | 0.00 | 1.06 | | 1.06 | 1.07 | 0.00 |
| C76-C80 | year | | 0.97 | 0.95 | 0.99 | 0.01 | 0.99 | | 0.96 | 1.01 | 0.26 |
| C76-C80 | age | | 1.11 | 1.10 | 1.13 | 0.00 | 1.12 | | 1.10 | 1.13 | 0.00 |
| C81 | year | | 1.01 | 1.00 | 1.02 | 0.16 | 1.01 | | 1.00 | 1.03 | 0.11 |
| C81 | age | | 1.02 | 1.02 | 1.03 | 0.00 | 1.01 | | 1.01 | 1.02 | 0.00 |
| C82-C88 | year | | 1.01 | 1.00 | 1.02 | 0.11 | 1.01 | | 0.99 | 1.02 | 0.39 |
| C82-C88 | age | | 1.06 | 1.05 | 1.06 | 0.00 | 1.07 | | 1.06 | 1.08 | 0.00 |
| C90 | year | | 1.01 | 0.98 | 1.04 | 0.57 | 1.00 | | 0.97 | 1.04 | 0.79 |
| C90 | age | | 1.19 | 1.15 | 1.23 | 0.00 | 1.19 | | 1.15 | 1.23 | 0.00 |
| C91-C95 | year | | 1.00 | 0.99 | 1.01 | 0.99 | 1.00 | | 0.99 | 1.01 | 0.78 |
| C91-C95 | age | | 1.00 | 1.00 | 1.01 | 0.28 | 1.00 | | 0.99 | 1.00 | 0.67 |
| C96 | year | | 1.05 | 0.99 | 1.12 | 0.10 | 1.07 | | 0.98 | 1.17 | 0.15 |
| C96 | age | | 1.04 | 1.01 | 1.07 | 0.01 | 1.05 | | 1.00 | 1.09 | 0.04 |
| **RR = relative risk; 95%-CI = 95% confidence interval* | | | | | | | | | | | |

Table S5: Results from the Poisson regression estimating trends in cancer mortality

|  |  | **Men** |  |  |  | **Women** |  |  |  |
| --- | --- | --- | --- | --- | --- | --- | --- | --- | --- |
| **ICD** |  | **RR** | **[95%-CI]** | | **p-value** | **RR** | **[95%-CI]** | | **p-value** |
| C00-C97 | year | 0.97 | 0.97 | 0.98 | 0.00 | 0.98 | 0.98 | 0.99 | 0.00 |
| C00-C97 | age | 1.11 | 1.11 | 1.12 | 0.00 | 1.12 | 1.12 | 1.12 | 0.00 |
| C00-C14 | year | 0.94 | 0.92 | 0.96 | 0.00 | 0.96 | 0.92 | 1.01 | 0.08 |
| C00-C14 | age | 1.24 | 1.21 | 1.28 | 0.00 | 1.18 | 1.12 | 1.23 | 0.00 |
| C15-C26 | year | 0.98 | 0.97 | 0.99 | 0.00 | 0.99 | 0.97 | 1.00 | 0.03 |
| C15-C26 | age | 1.18 | 1.16 | 1.19 | 0.00 | 1.16 | 1.14 | 1.17 | 0.00 |
| C30-C39 | year | 0.95 | 0.94 | 0.96 | 0.00 | 0.97 | 0.96 | 0.99 | 0.00 |
| C30-C39 | age | 1.24 | 1.22 | 1.26 | 0.00 | 1.22 | 1.19 | 1.24 | 0.00 |
| C40-C41 | year | 1.00 | 0.96 | 1.05 | 0.85 | 1.00 | 0.95 | 1.05 | 0.95 |
| C40-C41 | age | 1.01 | 0.99 | 1.03 | 0.30 | 1.01 | 0.99 | 1.03 | 0.43 |
| C43 | year | 0.99 | 0.96 | 1.02 | 0.37 | 0.99 | 0.96 | 1.02 | 0.57 |
| C43 | age | 1.12 | 1.10 | 1.15 | 0.00 | 1.12 | 1.09 | 1.15 | 0.00 |
| C45-C49 | year | 1.00 | 0.97 | 1.03 | 0.95 | 1.00 | 0.97 | 1.04 | 0.82 |
| C45-C49 | age | 1.05 | 1.03 | 1.06 | 0.00 | 1.05 | 1.03 | 1.07 | 0.00 |
| C50 | year | 0.96 | 0.84 | 1.09 | 0.53 | 0.98 | 0.97 | 0.99 | 0.00 |
| C50 | age | 1.14 | 1.03 | 1.27 | 0.02 | 1.17 | 1.16 | 1.18 | 0.00 |
| C51-C58 | year |  |  |  |  | 0.98 | 0.97 | 0.99 | 0.00 |
| C51-C58 | age |  |  |  |  | 1.15 | 1.13 | 1.16 | 0.00 |
| C60-C63 | year | 0.97 | 0.94 | 1.01 | 0.12 |  |  |  |  |
| C60-C63 | age | 1.09 | 1.06 | 1.11 | 0.00 |  |  |  |  |
| C64-C68 | year | 0.97 | 0.95 | 1.00 | 0.05 | 0.99 | 0.96 | 1.03 | 0.67 |
| C64-C68 | age | 1.16 | 1.13 | 1.20 | 0.00 | 1.11 | 1.08 | 1.14 | 0.00 |
| C69-C72 | year | 1.00 | 0.98 | 1.01 | 0.68 | 0.99 | 0.97 | 1.01 | 0.28 |
| C69-C72 | age | 1.05 | 1.05 | 1.06 | 0.00 | 1.05 | 1.04 | 1.06 | 0.00 |
| C73-C75 | year | 1.01 | 0.96 | 1.06 | 0.80 | 1.00 | 0.95 | 1.05 | 0.98 |
| C73-C75 | age | 1.02 | 1.00 | 1.04 | 0.13 | 1.02 | 1.00 | 1.05 | 0.05 |
| C76-C80 | year | 0.97 | 0.95 | 1.00 | 0.04 | 0.98 | 0.95 | 1.01 | 0.21 |
| C76-C80 | age | 1.11 | 1.09 | 1.13 | 0.00 | 1.12 | 1.09 | 1.14 | 0.00 |
| C81 | year | 0.95 | 0.89 | 1.02 | 0.18 | 0.92 | 0.85 | 1.01 | 0.07 |
| C81 | age | 1.07 | 1.03 | 1.11 | 0.00 | 1.05 | 1.01 | 1.09 | 0.02 |
| C82-C88 | year | 0.97 | 0.94 | 1.00 | 0.03 | 0.96 | 0.93 | 1.00 | 0.04 |
| C82-C88 | age | 1.08 | 1.06 | 1.10 | 0.00 | 1.07 | 1.05 | 1.10 | 0.00 |
| C90 | year | 0.98 | 0.92 | 1.04 | 0.43 | 0.96 | 0.89 | 1.03 | 0.28 |
| C90 | age | 1.24 | 1.14 | 1.34 | 0.00 | 1.22 | 1.11 | 1.34 | 0.00 |
| C91-C95 | year | 0.97 | 0.95 | 0.99 | 0.01 | 0.97 | 0.95 | 1.00 | 0.03 |
| C91-C95 | age | 1.03 | 1.02 | 1.04 | 0.00 | 1.03 | 1.02 | 1.04 | 0.00 |
| C96 | year | 1.00 | 0.77 | 1.29 | 0.99 | 1.04 | 0.80 | 1.35 | 0.78 |
| C96 | age | 1.04 | 0.92 | 1.16 | 0.55 | 1.00 | 0.89 | 1.11 | 0.93 |
| **RR = relative risk; 95%-CI = 95% confidence interval* | | | | | | | | | |

## Temporal trends in the incidence rate of early-onset cancers among men and women

Figure S1: Temporal trends in the incidence rate by early-onset cancer type by age among men


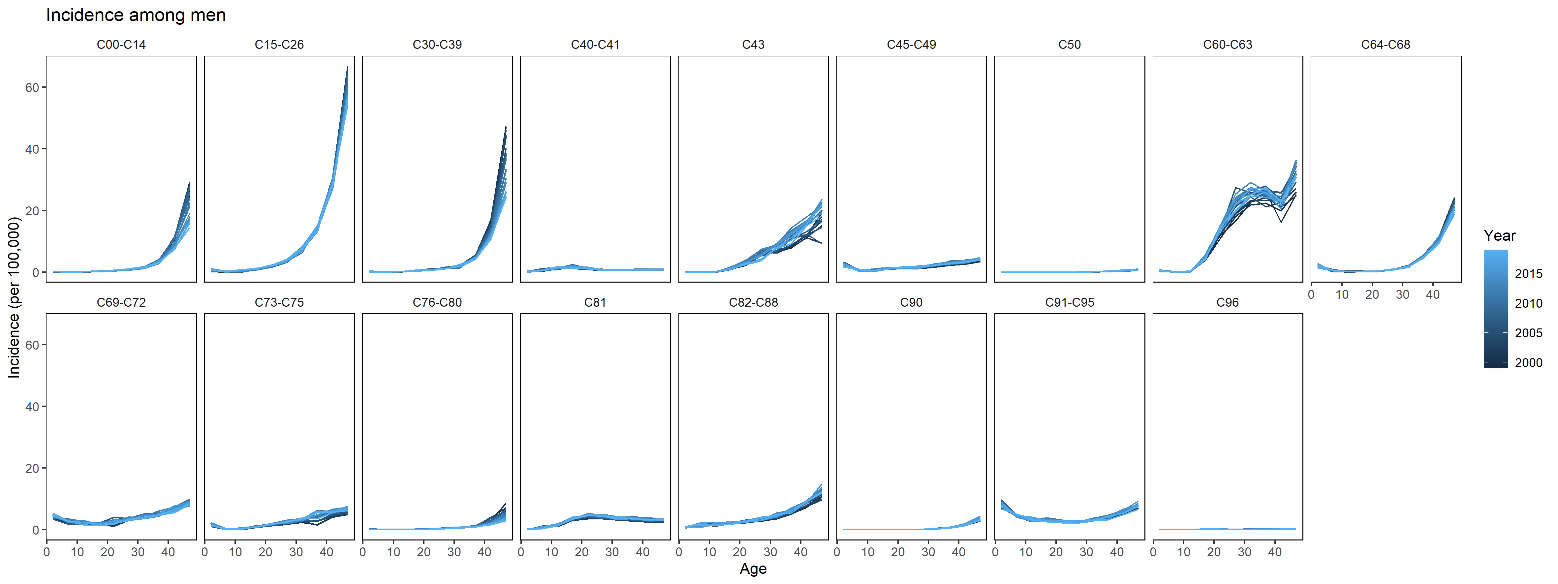


Figure S2: Temporal trends in the incidence rate by early-onset cancer type by age among women


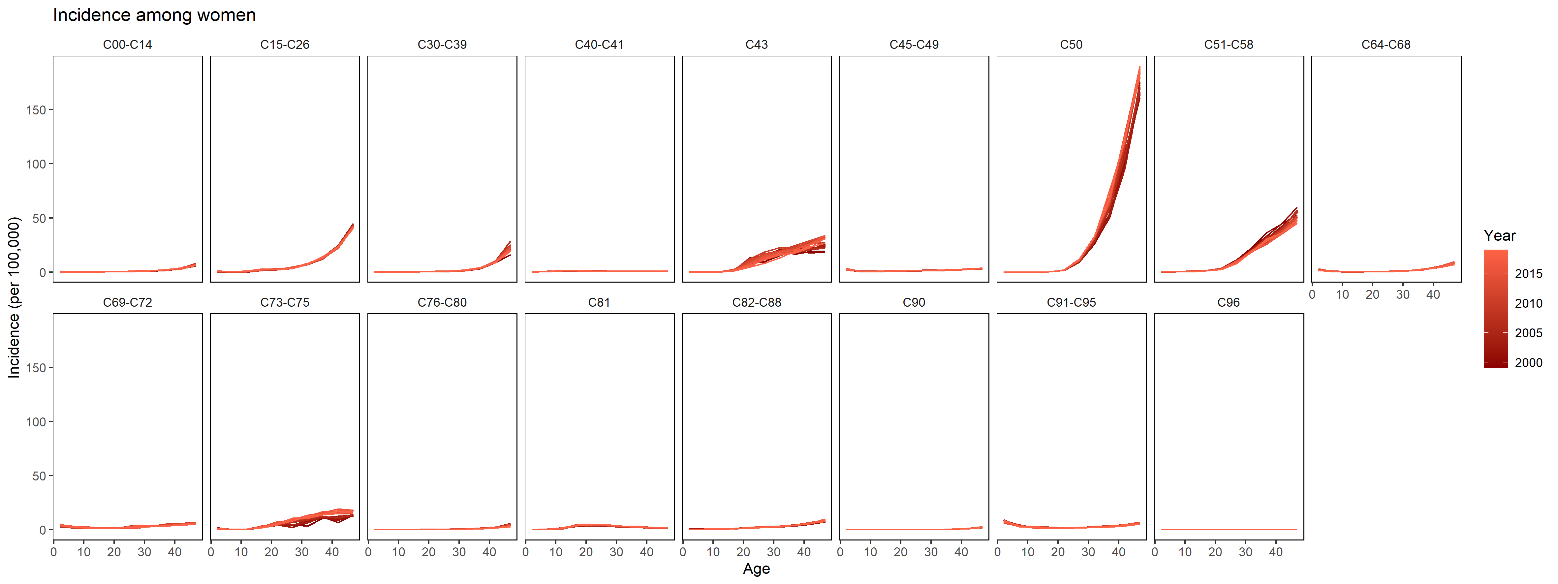


Figure S3: Temporal trends in the mortality rate by early-onset cancer type by age among men


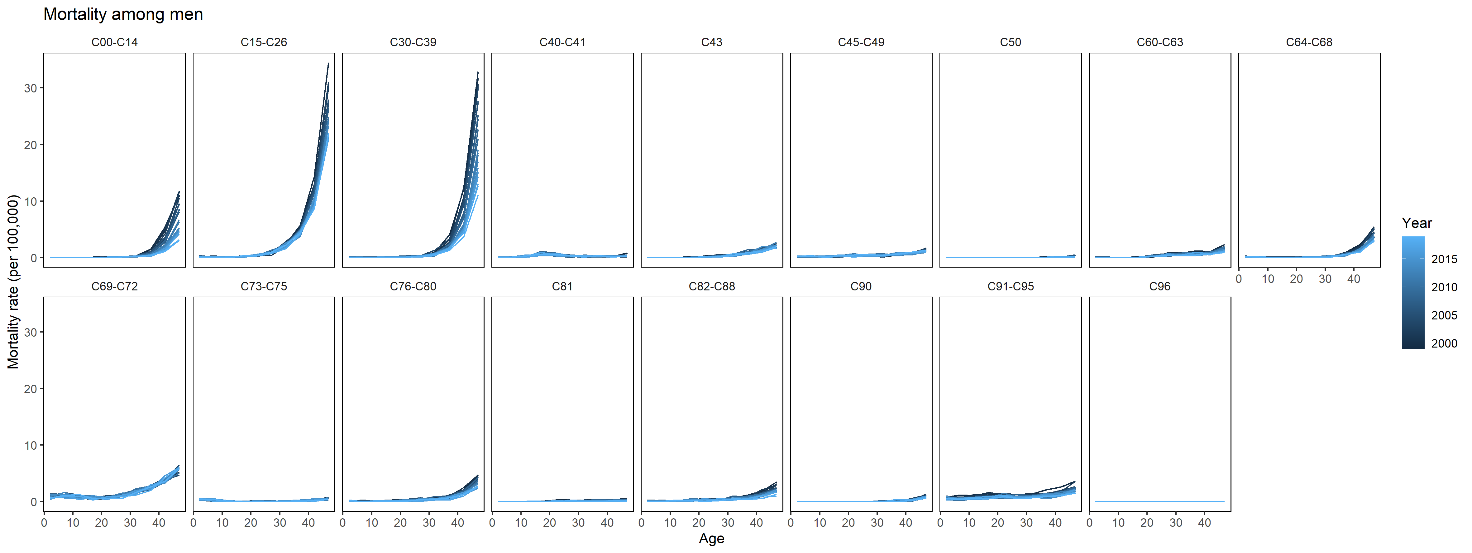


Figure S4: Temporal trends in the mortality rate by early-onset cancer type by age among women


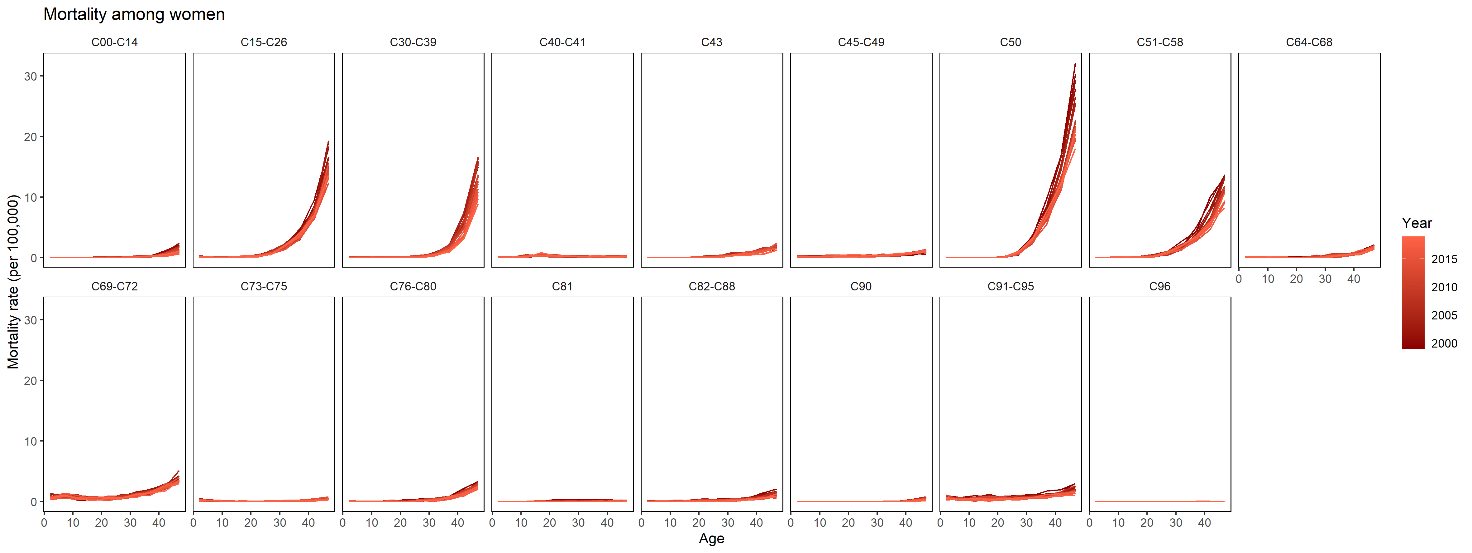


## Total Cancer (C00-C97) Incidence Development over Time

Figure S5: Temporal trend in the incidence of total early-onset cancer by age among men. Grey dashed lines are for reference and better comparability among the different figures only


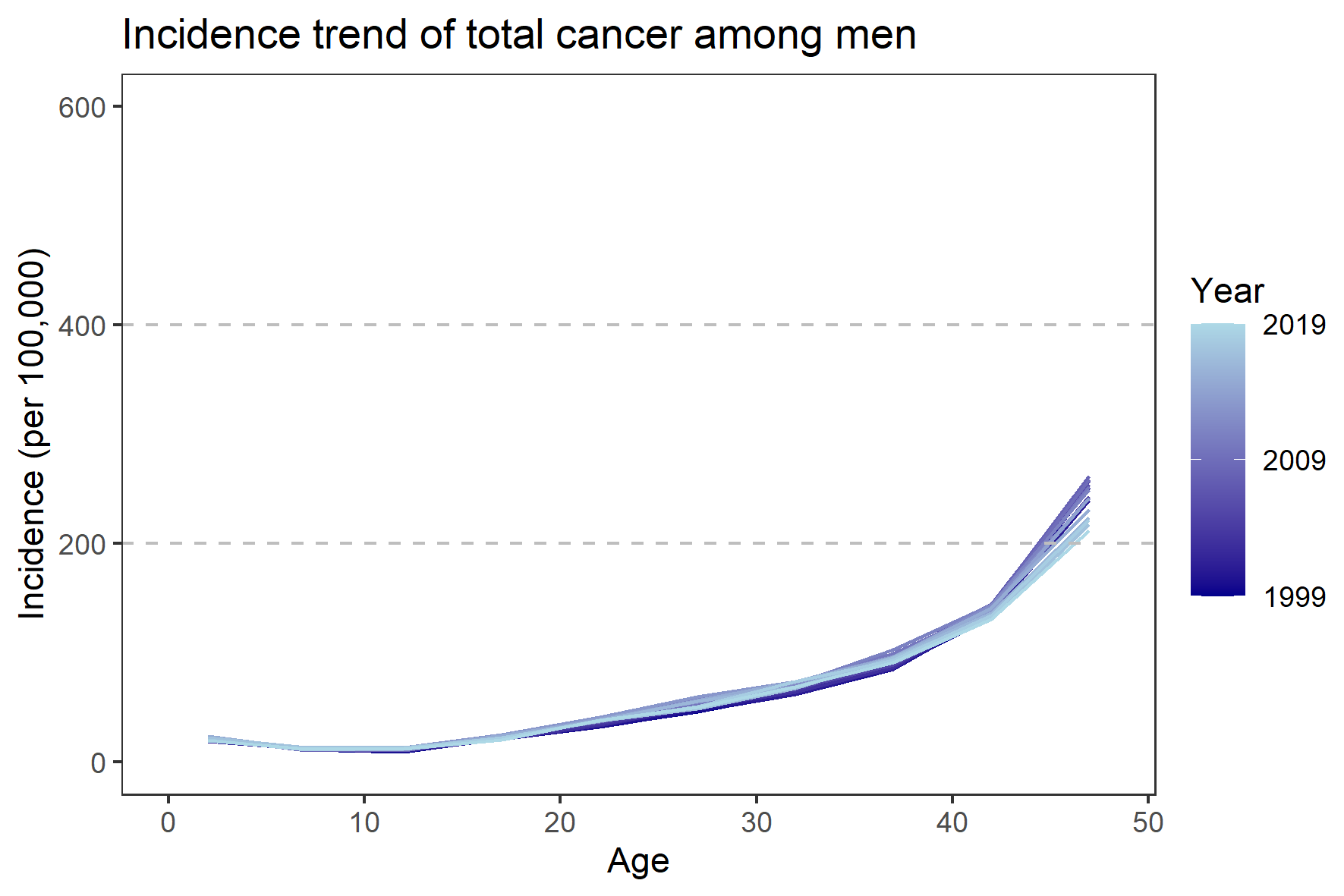


Figure S6: Temporal trend in the incidence of total early-onset cancer by age among women. Grey dashed lines are for reference and better comparability among the different figures only


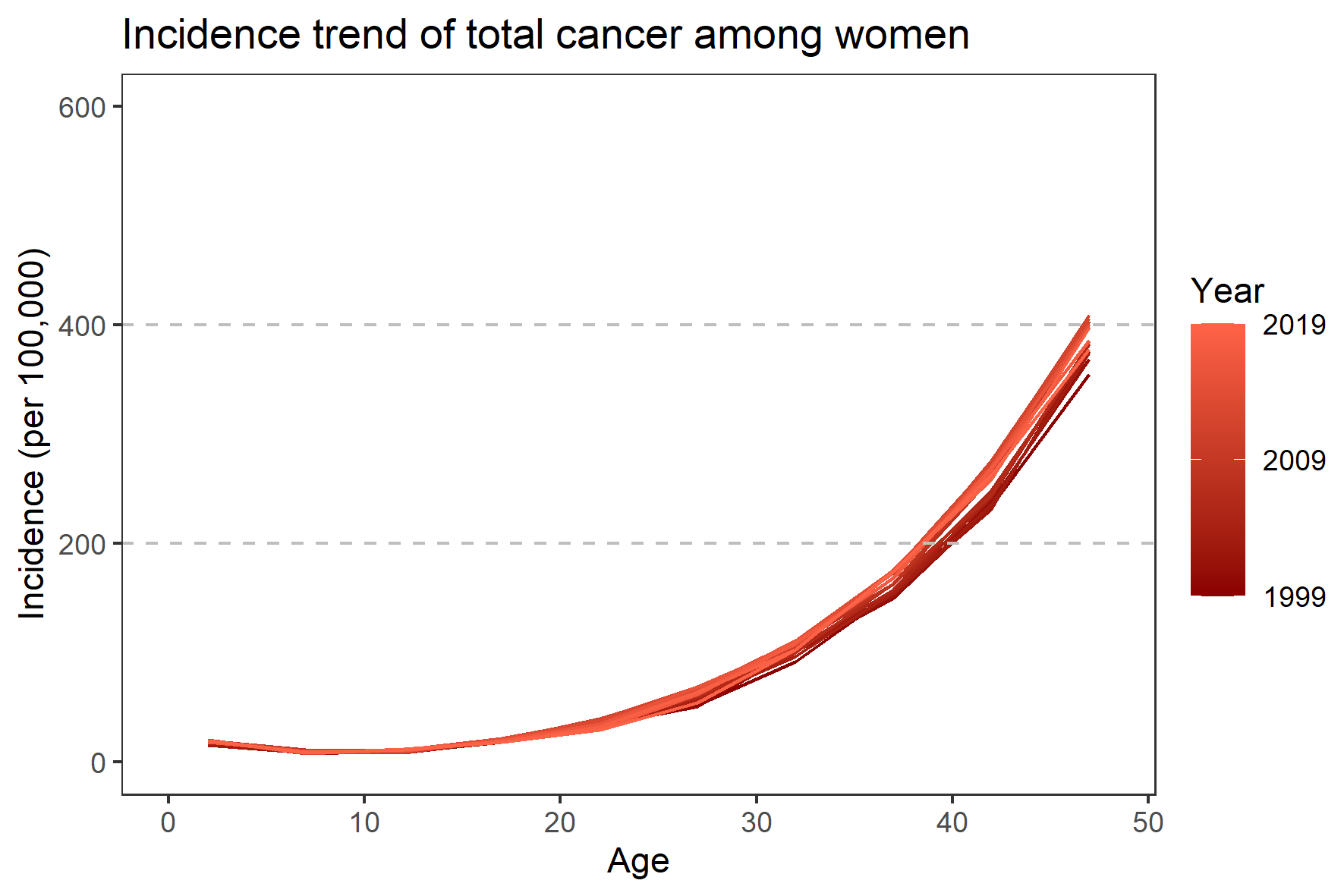


## Total Cancer (C00-C97) Mortality Development over Time

Figure S7: Temporal trend in the mortality rate of total early-onset cancer by age among men. Grey dashed lines are for reference and better comparability among the different figures only


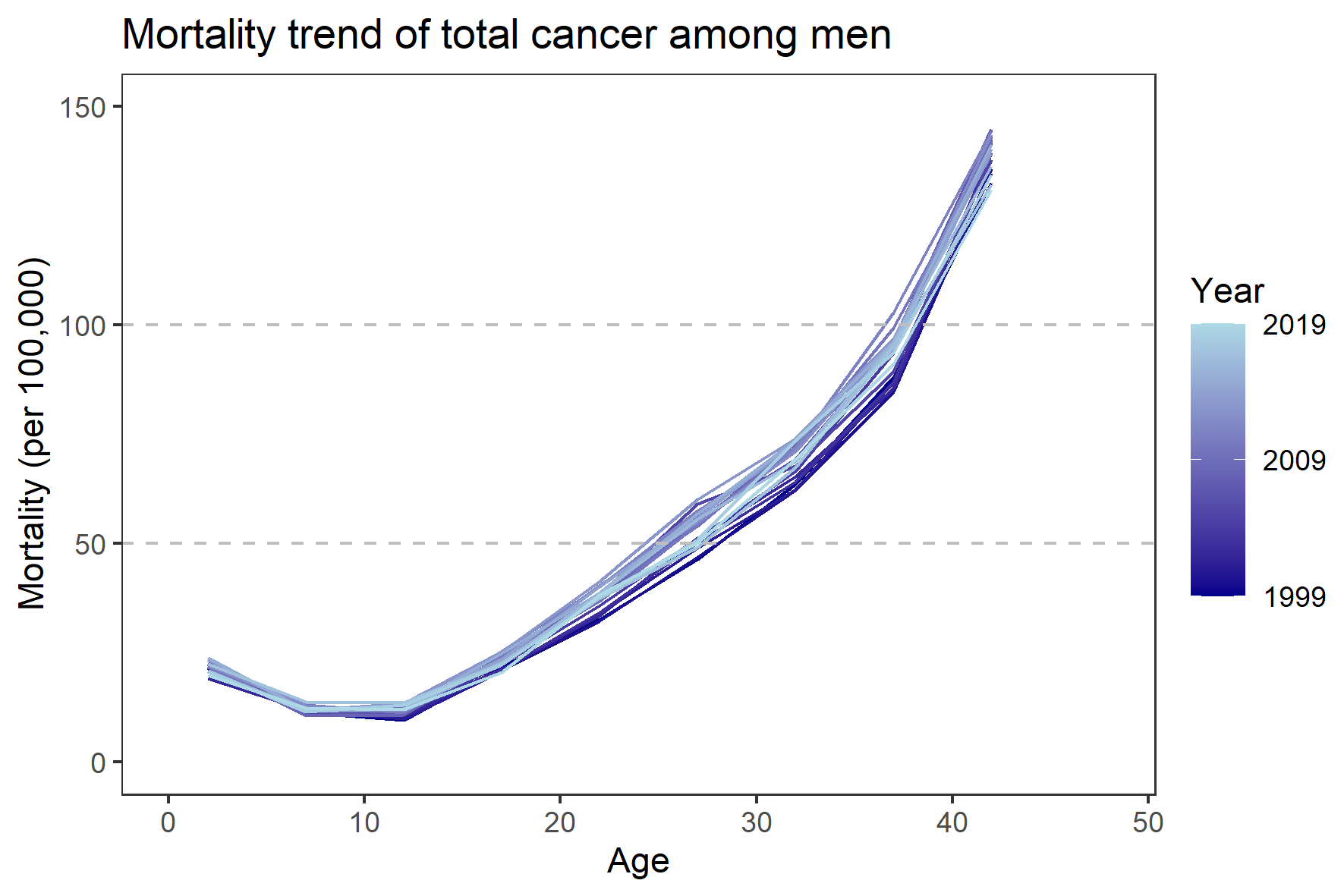


Figure S8: Temporal trend in the mortality rate of total early-onset cancer by age among women. Grey dashed lines are for reference and better comparability among the different figures only


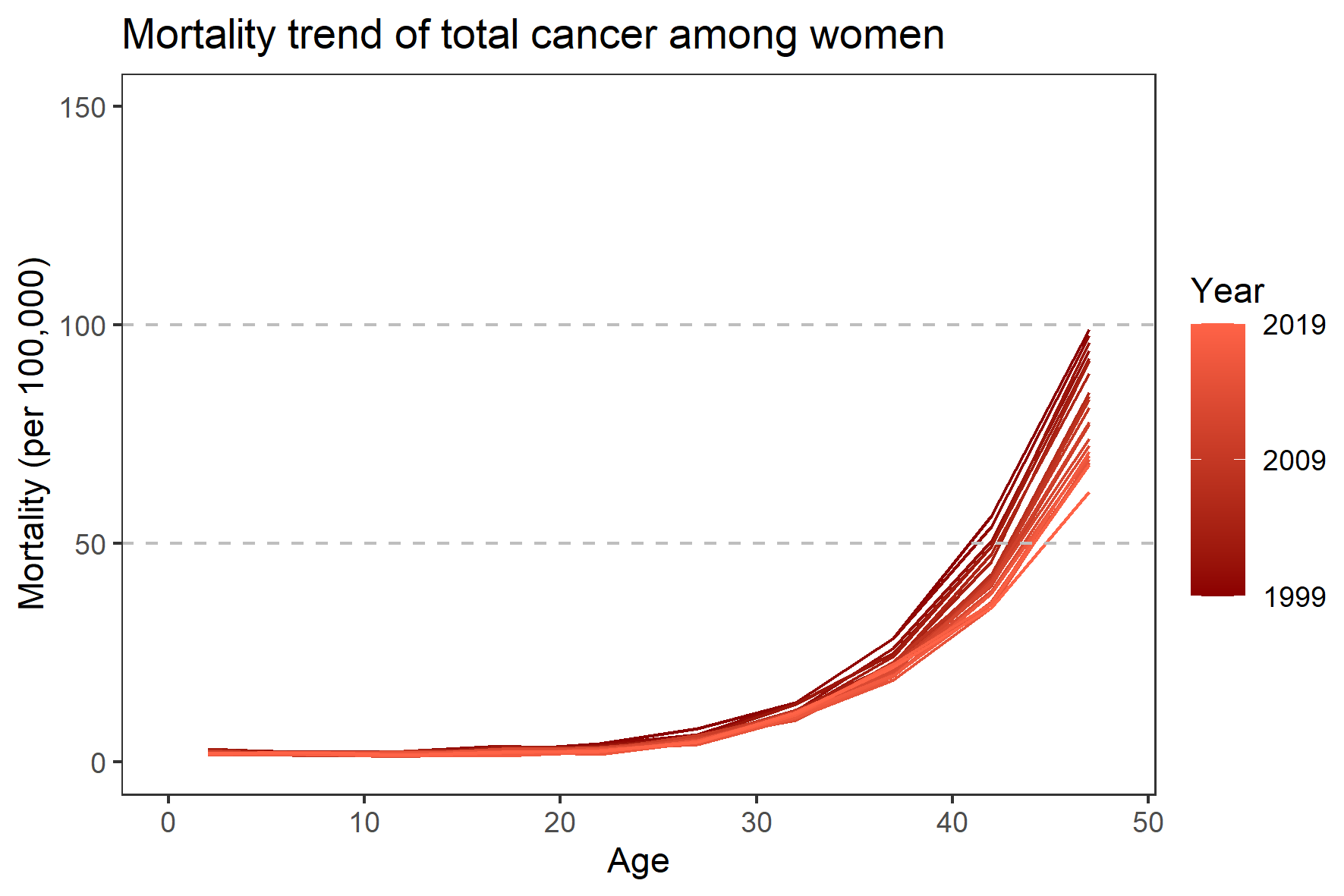


## Temporal Trend of the Total Cancer (C00-C97) Incidence by Age

Figure S9: Relation of the incidence of total early-onset cancer and age among men. Grey dashed lines are for reference and better comparability among the different figures only


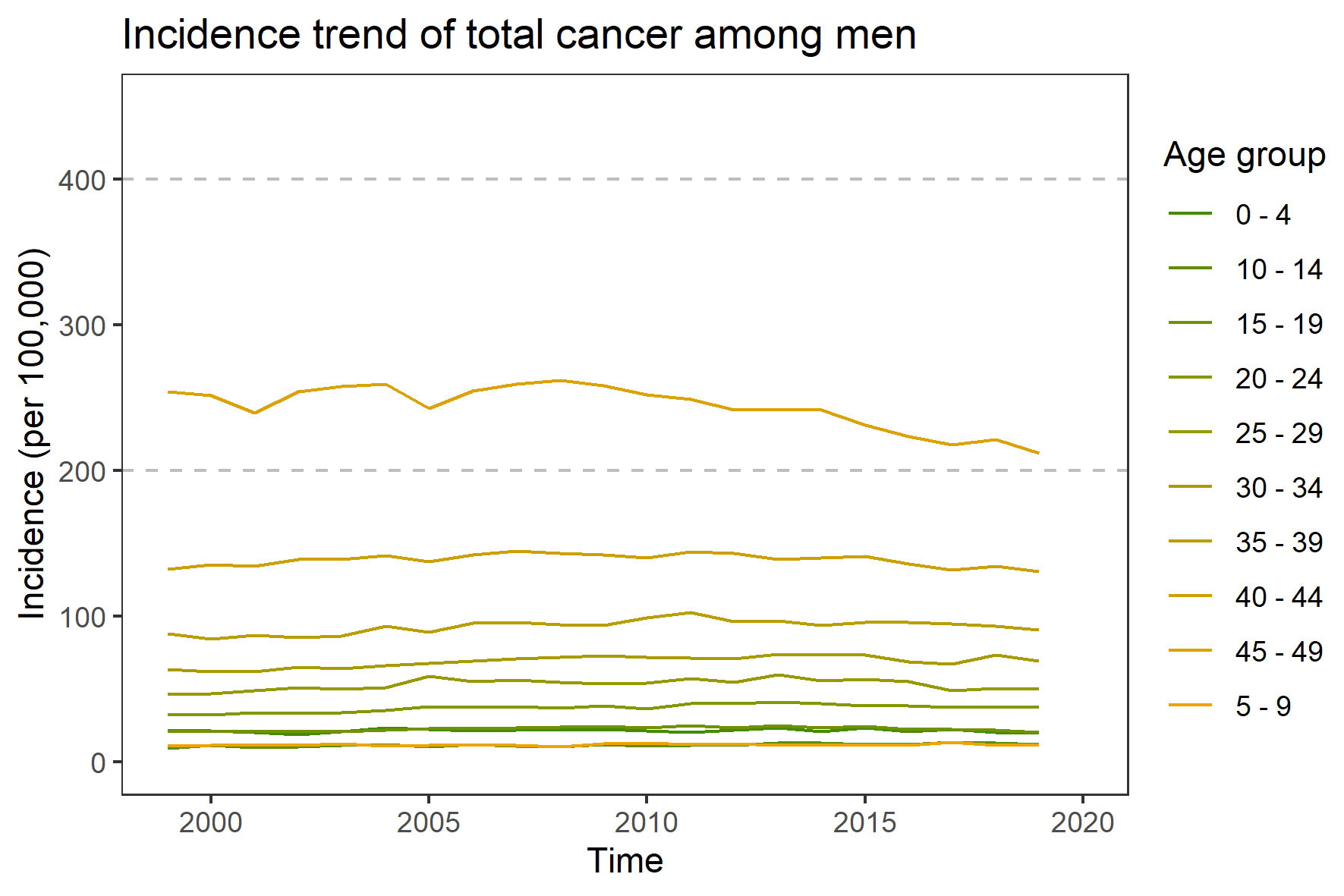


Figure S10: Relation of the incidence of total early-onset cancer and age among women. Grey dashed lines are for reference and better comparability among the different figures only


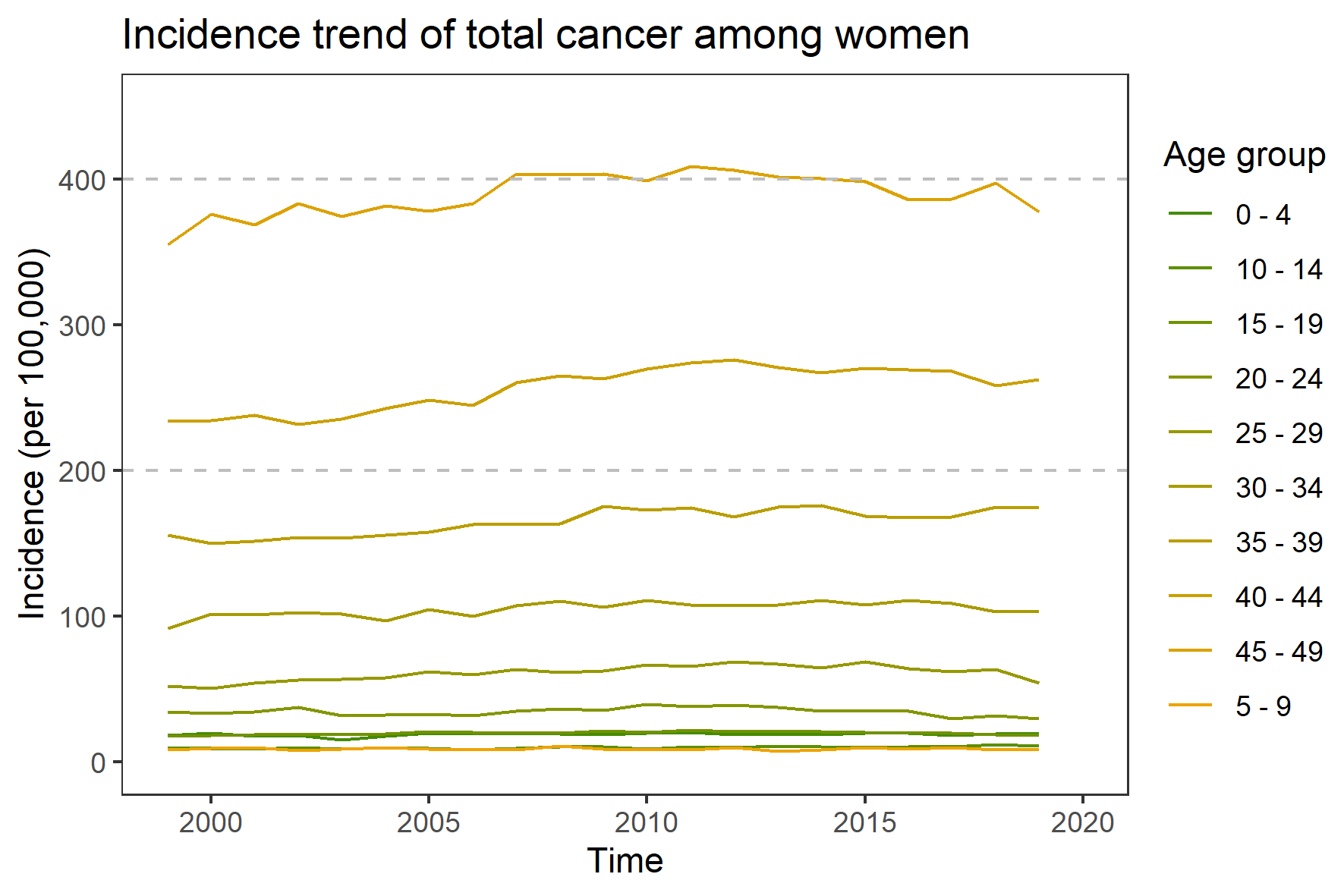


## Temporal Trend of the Total Cancer (C00-C97) Mortality by Age

Figure S11: Relation of the mortality of total early-onset cancer and age among men. Grey dashed lines are for reference and better comparability among the different figures only


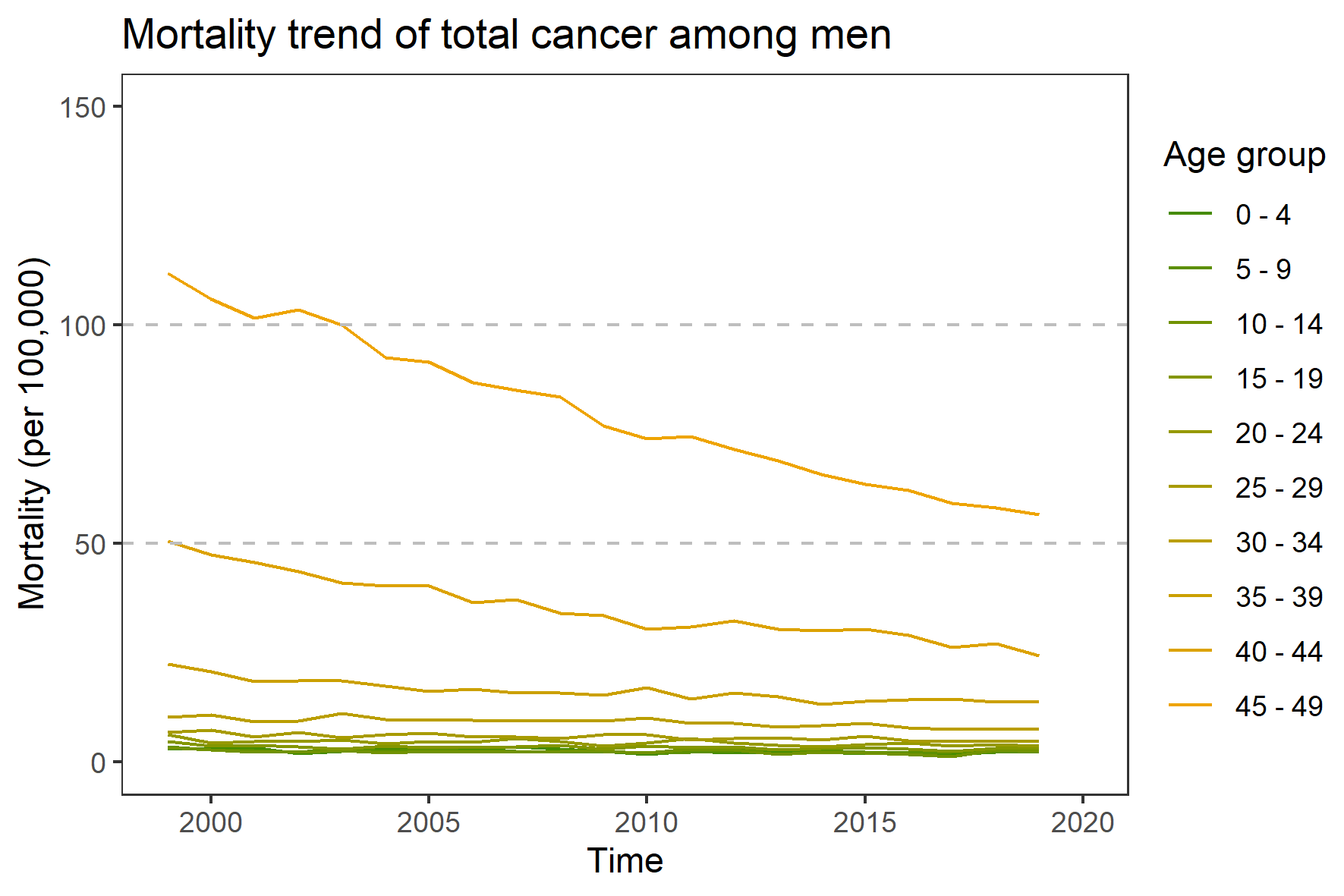


Figure S12: Relation of the mortality of total early-onset cancer and age among women. Grey dashed lines are for reference and better comparability among the different figures only. Grey dashed lines are for reference and better comparability among the different figures only


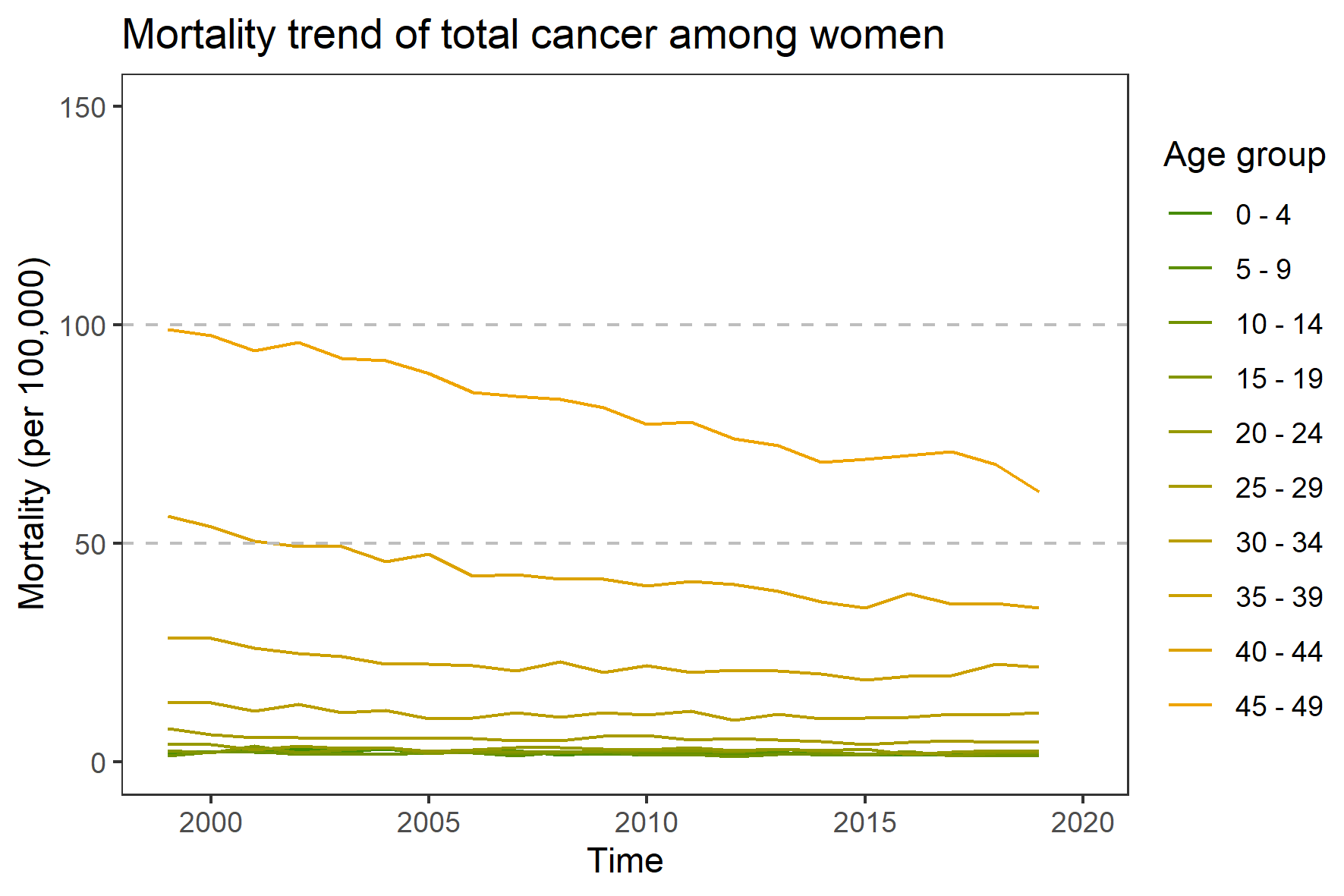


## Late-Onset Cancer (C00-C97) Incidence Development over Time

Figure S13: Incidence rate (per 100,000) of all late-onset cancers, i.e., ICD-10 C00-C97 excl. C44 diagnosed among individuals aged older than 50 years, combined between 1999 and 2019


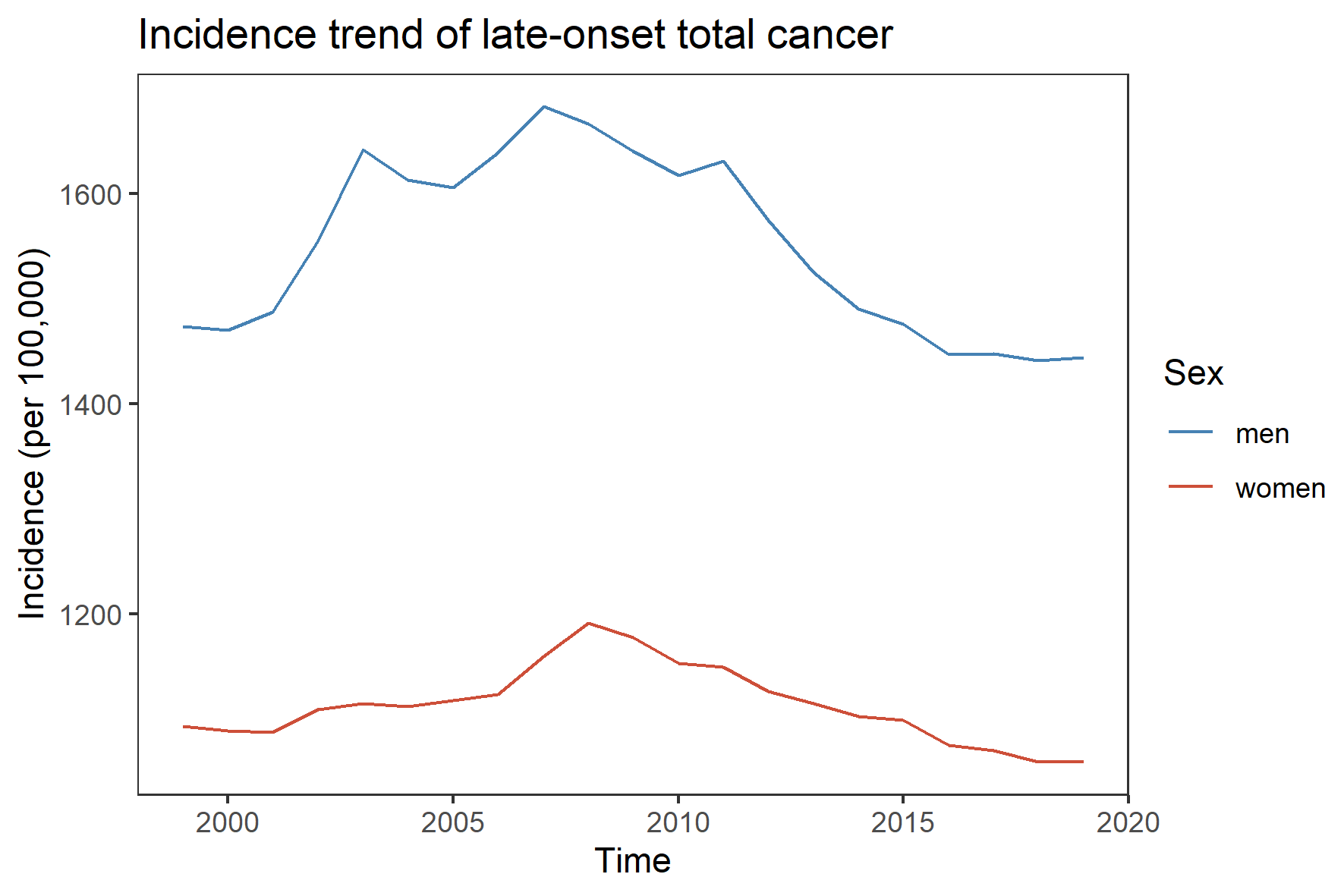


## Cancer registry completeness estimation

Table S6: Overview of all federal states in Germany and their data transmission to the registry between 1990 and 2019 provided by the ZfKD [1]

## Assessment of overdispersion

Table S7: Assessing potential over-/underdispersion using the function “dispersiontest()” from package AER (version 1.2-12) in R, which tests the null hypothesis of equidispersion in Poisson GLMs against the alternative of overdispersion and/or underdispersion. Overdispersion corresponds to α > 0 while underdispersion corresponds to α < 0.

| **Test for over-/underdispersion (assessed via R-function "dispersiontest()” from package AER)** | | | | | |
| --- | --- | --- | --- | --- | --- |
| **Cancer group (ICD 10)** |  | **Incidence (Men)** | **Incidence (Women)** | **Mortality (Men)** | **Mortality (Women)** |
| C00-C14 | p | 1.0000 | 1.0000 | 0.9936 | 0.9992 |
|  | α | -0.7152 | -0.0916 | -5.0538 | -1.2944 |
|  |  |  |  |  |  |
| C00-C97 | p | 0.0000 | 0.0000 | 0.0004 | 0.9996 |
|  | α | 2.9251 | 2.9491 | 1.3704 | 0.7190 |
|  |  |  |  |  |  |
| C15-C26 | p | 1.0000 | 1.0000 | 1.0000 | 1.0000 |
|  | α | -0.0241 | 0.1451 | -0.5902 | -0.2439 |
|  |  |  |  |  |  |
| C30-C39 | p | 1.0000 | 1.0000 | 1.0000 | 1.0000 |
|  | α | -0.8257 | -0.8936 | -4.9820 | -3.2941 |
|  |  |  |  |  |  |
| C40-C41 | p | 1.0000 | 1.0000 | 1.0000 | 1.0000 |
|  | α | 0.2587 | 0.1745 | 0.1515 | 0.0924 |
|  |  |  |  |  |  |
| C43 | p | 0.9982 | < 2.2e-16 | 1.0000 | 1.0000 |
|  | α | 0.8200 | 2.3157 | 0.2954 | 0.3157 |
|  |  |  |  |  |  |
| C45-C49 | p | 1.0000 | 1.0000 | 1.0000 | 1.0000 |
|  | α | 0.3921 | 0.3628 | 0.0341 | 0.0216 |
|  |  |  |  |  |  |
| C50 | p | 0.9735 | < 2.2e-16 | 0.9726 | 1.0000 |
|  | α | -0.1769 | 2.2957 | -2.6540 | 0.6352 |
|  |  |  |  |  |  |
| C51 – C58 (women)/ C60-C63 (men) | p | < 2.2e-16 | 0.0000 | 1.0000 | 1.0000 |
|  | α | 3.3610 | 1.3045 | 0.2248 | 0.2694 |
|  |  |  |  |  |  |
| C64-C68 | p | 0.0023 | 0.0635 | 1.0000 | 1.0000 |
|  | α | 1.9605 | 1.2273 | -3.2545 | -0.7019 |
|  |  |  |  |  |  |
| C69-C72 | p | 1.0000 | 1.0000 | 1.0000 | 1.0000 |
|  | α | 0.4493 | 0.3960 | 0.1411 | 0.1420 |
|  |  |  |  |  |  |
| C73-C75 | p | 1.0000 | 0.9942 | 1.0000 | 1.0000 |
|  | α | 0.3578 | 0.8397 | 0.1345 | 0.0779 |
|  |  |  |  |  |  |
| C76-C80 | p | 1.0000 | 1.0000 | 1.0000 | 1.0000 |
|  | α | -0.2703 | -0.1824 | -0.3008 | -0.4266 |
|  |  |  |  |  |  |
| C81 | p | 1.0000 | 0.4284 | 1.0000 | 1.0000 |
|  | α | 0.6307 | 1.0109 | 0.1915 | 0.1189 |
|  |  |  |  |  |  |
| C82-C88 | p | 1.0000 | 1.0000 | 1.0000 | 1.0000 |
|  | α | 0.0573 | 0.0625 | -0.0141 | 0.0161 |
|  |  |  |  |  |  |
| C90 | p | 1.0000 | 0.9954 | 0.9344 | 0.9526 |
|  | α | 0.3470 | -0.3917 | -0.3557 | -6.6682 |
|  |  |  |  |  |  |
| C91-C95 | p | 0.8701 | 0.4599 | 1.0000 | 1.0000 |
|  | α | 0.9256 | 1.0080 | 0.0608 | 0.0689 |
|  |  |  |  |  |  |
| C96 | p | 1.0000 | 1.0000 | 0.9996 | 1.0000 |
|  | α | 0.1196 | 0.0726 | -0.0176 | 0.0750 |

## Joinpoint Regression Output

Table S8: Results from the joinpoint regression estimating trends in cancer incidence between 1999 and 2019.

| **Sex** | **ICD-10** | **Description** | **No. of Joinpoints** | **Average Annual Percentage Change (AAPC)** | **Lower 95%-CI** | **Upper 95%-CI** | **Statistically Significant (0=No, 1=Yes)** | **P-Value** |
| --- | --- | --- | --- | --- | --- | --- | --- | --- |
| **Men** | C00 – C97 (excl. C44) | Total cancer | 2 | -0.11 | -0.29 | 0.06 | 0 | 0.17 |
|  | C00 – C14 | Malignant neoplasms of lip, oral cavity and pharynx | 1 | -1.97 | -2.17 | -1.75 | 1 | 0.00 |
|  | C15 – C26 | Malignant neoplasms of digestive organs | 2 | -0.37 | -0.64 | -0.11 | 1 | 0.02 |
|  | C30 – C39 | Malignant neoplasms of respiratory and intrathoracic organs | 1 | -2.34 | -2.59 | -2.09 | 1 | 0.00 |
|  | C40 – C41 | Malignant neoplasms of bone and articular cartilage | 0 | 0.10 | -1.15 | 1.34 | 0 | 0.86 |
|  | C43 | Melanoma and other malignant neoplasms of skin | 1 | 0.98 | 0.16 | 1.68 | 1 | 0.03 |
|  | C45 – C49 | Malignant neoplasms of mesothelial and soft tissue | 1 | 0.47 | -0.10 | 1.11 | 0 | 0.11 |
|  | C50 | Malignant neoplasm of breast | 0 | 1.00 | -1.12 | 3.10 | 0 | 0.34 |
|  | C60 – C63 | Malignant neoplasms of male genital organs | 2 | 0.73 | 0.33 | 1.12 | 1 | 0.00 |
|  | C64 – C68 | Malignant neoplasms of urinary tract | 3 | -0.69 | -1.06 | -0.41 | 1 | 0.00 |
|  | C69 – C72 | Malignant neoplasms of eye, brain and other parts of central nervous system | 1 | 0.04 | -0.25 | 0.31 | 0 | 0.78 |
|  | C73 – C75 | Malignant neoplasms of thyroid and other endocrine glands | 1 | 1.76 | 1.00 | 2.37 | 1 | 0.00 |
|  | C76 – C80 | Malignant neoplasms of ill-defined, secondary and unspecified sites | 1 | -2.99 | -3.70 | -2.29 | 1 | 0.00 |
|  | C81 | Hodgkin Lymphoma | 2 | 0.37 | -0.15 | 0.83 | 0 | 0.15 |
|  | C82 – C88 | Non-Hodgkin-Lymphoma | 3 | 0.30 | 0.05 | 0.57 | 1 | 0.02 |
|  | C90 | Multiple myeloma and malignant plasma cell neoplasms | 1 | 0.43 | -0.54 | 1.15 | 0 | 0.25 |
|  | C91 – C95 | Leukaemia | 1 | -0.20 | -0.76 | 0.19 | 0 | 0.30 |
|  | C96 | Malignant neoplasms of independent (primary) multiple sites | 0 | 4.98 | 3.15 | 6.80 | 1 | 0.00 |
| **Women** | C00 – C97 (excl. C44) | Total cancer | 1 | 0.46 | 0.32 | 0.59 | 1 | 0.00 |
|  | C00 – C14 | Malignant neoplasms of lip, oral cavity and pharynx | 1 | 0.96 | 0.61 | 1.34 | 1 | 0.00 |
|  | C15 – C26 | Malignant neoplasms of digestive organs | 2 | -0.11 | -0.27 | 0.05 | 0 | 0.14 |
|  | C30 – C39 | Malignant neoplasms of respiratory and intrathoracic organs | 1 | 0.80 | 0.14 | 1.55 | 1 | 0.03 |
|  | C40 – C41 | Malignant neoplasms of bone and articular cartilage | 0 | 0.07 | -1.59 | 1.68 | 0 | 0.91 |
|  | C43 | Melanoma and other malignant neoplasms of skin | 3 | 0.54 | 0.20 | 0.93 | 1 | 0.00 |
|  | C45 – C49 | Malignant neoplasms of mesothelial and soft tissue | 0 | 0.51 | -0.20 | 1.20 | 0 | 0.16 |
|  | C50 | Malignant neoplasm of breast | 1 | 1.05 | 0.86 | 1.24 | 1 | 0.00 |
|  | C51 – C58 | Malignant neoplasms of female genital organs | 2 | -1.15 | -1.35 | -0.97 | 1 | 0.00 |
|  | C64 – C68 | Malignant neoplasms of urinary tract | 2 | -0.77 | -1.10 | -0.45 | 1 | 0.00 |
|  | C69 – C72 | Malignant neoplasms of eye, brain and other parts of central nervous system | 1 | -0.04 | -1.29 | 1.58 | 0 | 0.98 |
|  | C73 – C75 | Malignant neoplasms of thyroid and other endocrine glands | 2 | 2.34 | 1.67 | 3.10 | 1 | 0.00 |
|  | C76 – C80 | Malignant neoplasms of ill-defined, secondary and unspecified sites | 1 | -1.55 | -2.32 | -0.77 | 1 | 0.00 |
|  | C81 | Hodgkin Lymphoma | 0 | 1.23 | 0.68 | 1.78 | 1 | 0.00 |
|  | C82 – C88 | Non-Hodgkin-Lymphoma | 1 | 0.58 | 0.11 | 1.01 | 1 | 0.02 |
|  | C90 | Multiple myeloma and malignant plasma cell neoplasms | 1 | 0.45 | -0.65 | 1.62 | 0 | 0.33 |
|  | C91 – C95 | Leukaemia | 1 | -0.20 | -0.72 | 0.27 | 0 | 0.34 |
|  | C96 | Malignant neoplasms of independent (primary) multiple sites | NA | NA | NA | NA | 1 | NA |

References

1. Centre for Cancer Registry Data (ZfKD). Database: Cancer data up to 2019. https://www.krebsdaten.de/Krebs/EN/Home/homepage_node.html. Accessed 18 Sep 2023.

2. Arndt V. Cancer survivorship in Germany—epidemiology and definitions. In: ; 2019. p. 158–164.
